# Expression and Anthocyanin Biosynthesis-Modulating Potential of Sweet Cherry (*Prunus avium* L.) MYB10 and bHLH Genes

**DOI:** 10.1371/journal.pone.0126991

**Published:** 2015-05-15

**Authors:** Pavel Starkevič, Jurgita Paukštytė, Vaiva Kazanavičiūtė, Erna Denkovskienė, Vidmantas Stanys, Vidmantas Bendokas, Tadeušas Šikšnianas, Aušra Ražanskienė, Raimundas Ražanskas

**Affiliations:** 1 Vilnius University Institute of Biotechnology, V.A. Graičiūno 8, Vilnius, LT-02241, Lithuania; 2 Institute of Horticulture, Lithuanian Research Centre for Agriculture and Forestry, Kaunas st 30, Babtai, LT-54333, Kaunas, Lithuania; Zhejiang University, CHINA

## Abstract

Anthocyanins are essential contributors to fruit coloration, an important quality feature and a breed determining trait of a sweet cherry fruit. It is well established that the biosynthesis of anthocyanins is regulated by an interplay of specific transcription factors belonging to MYB and bHLH families accompanied by a WD40 protein. In this study, we isolated and analyzed *PaWD40*, *PabHLH3*, *PabHLH33*, and several closely related MYB10 gene variants from different cultivars of sweet cherry, analyzed their expression in fruits with different anthocyanin levels at several developmental stages, and determined their capabilities to modulate anthocyanin synthesis in leaves of two *Nicotiana* species. Our results indicate that transcription level of variant *PaMYB10*.*1-1* correlates with fruit coloration, but anthocyanin synthesis in *Nicotiana* was induced by another variant, *PaMYB10*.*1-3*, which is moderately expressed in fruits. The analysis of two fruit-expressed bHLH genes revealed that *PabHLH3* enhances MYB-induced anthocyanin synthesis, whereas *PabHLH33* has strong inhibitory properties.

## Introduction

**Fig 1:**
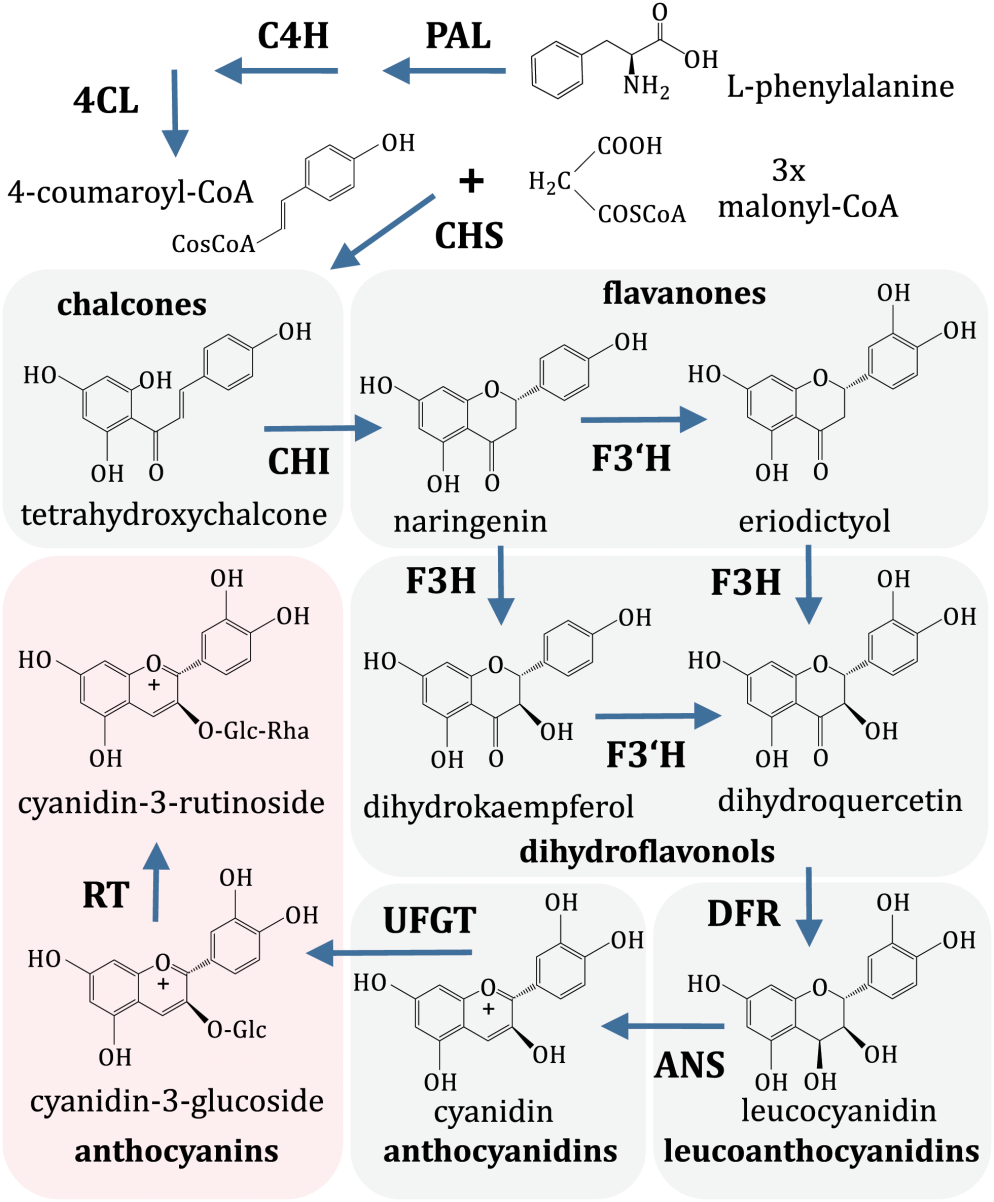
Anthocyanin pathway in sweet cherry. Only branch leading to the most prevalent cherry anthocyanin, cyanidin-3-rutinoside, is shown. Enzyme abbreviations: PAL, phenylalanine ammonia-lyase; C4H, cinnamate 4-hydroxylase; 4CL, 4-coumarate:CoA ligase; CHS, chalcone synthase; CHI, chalcone isomerase; F3H, flavanone 3-hydroxylase; F3’H, flavanone 3’-hydroxylase; DFR, dihydroflavonol reductase; ANS anthocyanidin synthase; UFGT, UDP-glucose:flavonoid 3-*O*-glucosyl transferase; RT, rhamnosyltransferase.

Sweet cherry (*Prunus avium* L.) is an important crop valued primarily for the fleshy fruits, an excellent source of many nutrients and phytochemicals. There are hundreds of sweet cherry varieties with fruit skin and flesh colors ranging from dark red to pale yellow, and this important quality is determined primarily by the accumulation of red anthocyanin pigments. Anthocyanins belong to flavonoids, an important group of plant secondary metabolites possessing antioxidant activity and other health-promoting qualities [1]. The general pathway of flavonoid synthesis is already well established in a model plant *Arabidopsis thaliana* [2] and has been studied in important fruit crops such as grapevine (*Vitis vinifera*) [3] and apple (*Malus domestica*) [4]. The flavonoid pathway branches from the general phenylpropanoid pathway when enzyme chalcone synthase (CHS) generates tetrahydroxychalcone from 4-coumaroyl-CoA and malonyl-CoA, which later is converted into flavanones, dihydroxyflavonols, and finally anthocyanins are synthesized [5]. The most abundant anthocyanins in cherry fruits are cyanidins, mainly cyanidin-3-rutinoside [6] (Fig 1).

Until recently, there has been no data about anthocyanin pathway genes and their regulation in *P*. *avium* and the whole *Prunus* genus, but the situation is changing rapidly. The sequencing of *P*. *persica* [7] and *P*. *mume* [8] genomes facilitates identification and analysis of genes not only in these species, but also greatly simplifies the cloning and analysis of genes of interest also in other species of the genus. Recently, anthocyanin pathway genes were investigated in nectarine [9], different cultivars of peach [10–12], both belonging to the same species *P*. *persica*, and sweet cherry [13, 14]. In general, anthocyanin synthesis genes in *Prunus* appeared to be closely related to their counterparts in species of other genera, especially the members of the Rosaceae family. The anthocyanin content in fruits of all investigated species closely correlated with the expression of at least some enzymes of the flavonoid pathway, especially participating in the final stages of anthocyanin production—UDP-glucose:flavonoid 3-*O*-glucosyltransferase (UFGT) and anthocyanidin synthase (ANS, also known as leucoanthocyanidin dioxygenase, LDOX). However, the regulation of anthocyanin biosynthesis is complex and variable.

The current understanding suggests that MYB-bHLH-WD40 (MBW) complexes are essential regulators of the flavonoid pathway. Various MBW complexes, differentially regulating the synthesis of anthocyanins and other flavonoids in different plant tissues and at different developmental stages, are assembled when constant member of the complex WD40 protein associates with different members of MYB and bHLH families. Thus, the DNA-binding specificity or other properties of available MYB and bHLH proteins determine the subset of genes that are activated in a given tissue or cell [15, 16]. Plant MYB proteins are the most plentiful and the most variable members of the complex participating in the regulation of secondary metabolism, signal transduction, development, and other pathways. These proteins contain single or multiple repeats comprised of structurally conserved DNA-binding domains [17]. The R2R3 MYB genes with two such repeats are the most abundant class with 126 members in *Arabidopsis*, and 13 of them are involved in the regulation of flavonoid metabolism [18]. Research on fruit crops identified that there are several MYB genes specifically responsible for the upregulation of anthocyanin biosynthesis genes in fruit tissues.

The first such gene identified in Rosaceae family was apple *MdMYB10* [4, 19, 20]. Soon after that, an array of homologous genes was cloned from different members of Rosaceae, and they all were named MYB10 implying their involvement in the regulation of anthocyanin biosynthesis [21]. Among these MYB10 genes, there were also genes from *P*. *avium*, *P*. *persica* and other members of the *Prunus* genus. Later, the expression and functional capabilities of the same MYB10 gene from *P*. *persica* was analyzed in nectarine [9] and blood-flesh peach [11]. However, another research identified that peach genome contains three very similar MYB10-like genes located close to each other and found that expression of previously analyzed variant of MYB10 gene (named *PpMYB10*.*2*) was barely detectable in fruits. Furthermore, functional tests and high level of expression in anthocyanin-rich tissues suggested that another MYB10 gene variant (named *PpMYB10*.*1*) is the most probable candidate for the fruit anthocyanin regulator [10]. Similarly, the expression of first isolated sweet cherry MYB10 gene correlated with anthocyanin production in fruits [21], but another study focused on another closely related MYB10 gene variant and also established that its expression correlated with anthocyanin content [13].

The second interchangeable constituent of the MBW complex is a member of a bHLH family, but the variety of flavonoid-regulating bHLH proteins seems to be much smaller when compared to MYBs—in *Arabidopsis* such functionality is assigned essentially to the TT8, EGL3 and GL3 [22]. Two distantly homologous counterparts of these proteins in apple [19] and later in strawberry [23] were named bHLH3 and bHLH33. The bHLH genes are considered to have partially overlapping expression patterns and have partially redundant roles in the regulation of anthocyanin biosynthesis in *Arabidopsis* seedlings [22, 24], but their expression and functional capabilities were analyzed in much lower extent in other species. In *Prunus*, bHLH3 has been isolated and analyzed in nectarine [9], whereas bHLH (and WD40) genes of *P*. *avium* have not been cloned and analyzed until now.

In this study, we cloned and sequenced anthocyanin pathway genes and their regulators *PaMYB10*, *PabHLH3*, *PabHLH33* and *PaWD40* from several *P*. *avium* cultivars and analyzed their expression in fruits of four cultivars with different anthocyanin synthesis patterns at five maturation time points. We resolved the ambiguity concerning expression levels of two previously analyzed MYB10 gene variants, isolated and sequenced previously unknown variants from several cultivars, and analyzed their expression and functional capabilities. We also analyzed data provided by the fundamental RNA sequencing (RNA-Seq) study of sweet cherry fruit exocarp development [25] and compared the expression of the same genes with our qPCR data.

## Materials and Methods

### Plant material

Fruits and leaves of *Prunus avium* cultivars ‘Kitayanka’, ‘Irema BS’, ‘Werdersche braune’, ‘Belobokaya rannyaya’ and sour cherry (*P*. *cerasus* L.) cultivar ‘Balaton’ were collected from adult trees during the 2012 season. Trees were grown at the Babtai Institute of Horticulture of Lithuanian Research Centre for Agriculture and Forestry under field conditions (lat: 55.0750, lng: 23.8100; elevation: 126 m). Three biological replicates, each containing 1 g of material from three fruits, were sampled from each analyzed sweet cherry cultivar from May to July at two-week intervals. All samples were immediately frozen in liquid nitrogen and stored at –80°C for subsequent analysis. For transient transformation experiments, *Nicotiana tabacum* and *N*. *benthamiana* were used.

### Measurement of total anthocyanin concentration

Anthocyanins were extracted from frozen berries grinded to a fine powder using 90% aqueous methanol, acidified with 0.02% HCl at a 1:20 g/ml ratio, and concentrated in a rotary evaporator at 37°C; residues were dissolved in acidified water (pH 2.4). The anthocyanin content was determined using a spectrophotometric differential pH method [26]. The absorbance of extracts was measured at 520 and 700 nm with a UV-VIS spectrophotometer Specord 210 PLUS (Analytik Jena AG) for 30 min at 25°C in the dark. The anthocyanin content was calculated using the molar extinction coefficient of cyanidin-3-glucoside chloride (c3g). Results were expressed as cyanidin-3-glucoside equivalents in mg per 100 g of fresh berry weight.

### DNA isolation, PCR, gene cloning and sequencing

DNA for gene cloning was isolated by GeneJET Plant Genomic DNA Purification Mini Kit (Thermo Fisher Scientific Baltics). Gene fragments or full genes were isolated by PCR using primers specific to known *P*. *avium* sequences or partly degenerated, designed to match the consensus sequence derived by aligning homologous *P*. *persica* sequences from NCBI whole genome sequence (WGS) database and any other homologous *Prunus* sequences available in public databases. All MYB10 gene variants from different *P*. *avium* cultivars were obtained by PCR using the same pair of primers PrPro_99-72d and PrCDS_2223-2196r (sequences of all cloning primers are presented in S1 Table). PCR reactions were accomplished using Phusion High-Fidelity DNA Polymerase (Thermo Fisher Scientific Baltics) with typical cycling parameters 98°C for 30 s, 40 cycles at 98°C for 15 s, 54°C for 30 s, 72°C for 90 s, and additional step at 72°C for 10 min. PCR fragments were cloned into vector pJET1.2-blunt (Thermo Fisher Scientific Baltics) and sequenced with capillary genetic analyzer 3130xl (Applied Biosystems).

### RNA isolation, cDNA synthesis and RACE

Pieces of three berries containing mesocarp and exocarp tissue were grinded in liquid nitrogen and isolated using Sigma Spectrum Plant Total RNA Kit (Sigma-Aldrich). cDNA synthesis and subsequent 5’-RACE was accomplished using RevertAid H Minus First Strand cDNA Synthesis Kit (Thermo Fisher Scientific Baltics). For 3’-RACE, cDNA was synthesized using primer 3-RACE-CDS-A, whereas cDNA for 5’-RACE and qPCR was synthesized using primer HOUSE_A. Template-switching reaction for 5’-RACE was performed with SMART-C3 oligo by using modified Clontech SMART protocol and Thermo Fisher Scientific Baltics enzymes and buffers [27]. Nonspecific (to cloned genes) primers for 3’-RACE and 5’-RACE were UPM and NUP-A, whereas specific primers were designed as already described. Cycling parameters for RACE PCR were: 98°C for 30 s; 5 cycles at 98°C for 15 s and at 72°C for 90 s; 5 cycles at 98°C for 15 s, at 69°C for 20 s, and at 72°C for 90 s; 30 cycles at 98°C for 15 s, at 66°C for 30 s, and at 72°C for 90 s; a final elongation at 72°C for 10 min.

### Gene expression analysis

Quantitative real-time PCR (qPCR) amplification was carried out using the Rotor Gene Q system (Qiagen). All reactions were performed in 25 μl volume with Maxima SYBR Green/ROX qPCR Master Mix (Thermo Fisher Scientific Baltics) using following cycling parameters: 10 min at 95°C; 40 cycles for 15 s at 95°C; 60 s at 60°C. The reactions were followed by melting curve detection gradient from 60°C to 95°C. All reactions were run in triplicate, and resulting data analyzed with Rotor-Gene Q Series software version 2.1.0 using ΔΔCt method and taking into account fragment replication efficiency in each experiment (formula N^-ΔΔCt^ was used, where replication efficiency N was typically about 1.8 per cycle). The expression level was normalized to *P*. *avium* actin variant 1 which, according to RNA-Seq data, is expressed evenly and at high level throughout all stages of fruit growth. The expression of enzyme genes *PaPAL*, *PaCHS*, *PaCHI*, *PaF3H*, *PaF3’H*, *PaDFR*, *PaANS*, *PaUFGT* and their putative regulators *PaWD40*, *PabHLH33*, *PabHLH3* along with different variants of MYB10 was analyzed using the same pair of gene-specific primers for each cultivar. All qPCR primer sequences are listed in S2 Table.

### Construction of plasmids for transient gene expression assays

*PaWD40*, *PabHLH3*, *PabHLH33*, *PaMYB10*.*1–1*, *PaMYB10*.*1–*2 and *PaMYB10*.*1–3* genes were PCR amplified using primers listed in S3 Table by Taq or Phusion polymerase (Thermo Fisher Scientific Baltics) and cloned into pTZ57R/T or pJET1.2 vectors, respectively. *Nru*I/*Not*I (*PaWD40*, *PabHLH33* and MYB‘s) or *Pvu*II/*Not*I (*PabHLH3*) digested fragments were inserted into *Smi*I/*Not*I digested vector pENTR/D (Invitrogen, Life technologies). *SlANT1* gene was PCR amplified from RNA isolated from cold-treated tomato “MicroTom” by using RevertAid H Minus First Strand cDNA Synthesis Kit (Thermo Fisher Scientific Baltics) and cloned into pTZ57R/T vector. *Sma*I/*Ecl*136II digested fragment was inserted into *Smi*I digested pENTR/D vector. *AtPAP1* gene was amplified from RNA isolated from cold-treated *A*. *thaliana* Col-0 plant using RevertAid H Minus First Strand cDNA Synthesis Kit and cloned into pTZ57R/T vector. *Smi*I/*Not*I digested fragment was inserted into *Smi*I digested pENTR/D vector. Gateway LR clonase II enzyme mix (Invitrogen, Life technologies) was used to recombine genes from entry plasmid into the final destination vector pAUGLR. Final plasmids were transformed into *Agrobacterium tumefaciens* GV3101 strain by electroporation.

### Transient gene expression assays

Wild type *N*. *tabacum* and *N*. *benthamiana* plants were grown in soil in an environment-controlled room at 24°C-25°C. All plants were maintained under long day conditions of 16 h white light and 8 h dark. Five-six week-old plants were syringe-infiltrated with *A*. *tumefaciens* suspension in abaxial side of the leaf. *A*. *tumefaciens* overnight cultures were grown in LB medium with appropriate antibiotics, diluted 500 times in LB containing 10 mM MES (2-(*N*-morpholino)ethanesulfonic acid) pH 5.7, and grown overnight again till A_600_ = 1.2–1.6. Cells then were sedimented by centrifugation for 20 min at 3000 g, suspended in infiltration buffer (10 mM MES pH 5.7, 10 mM MgCl_2_, 500 μM acetosyringone) till A_600_ = 1.8, and incubated overnight at room temperature on the table.

For infiltration, a helper strain containing the silencing suppressor from Plum pox virus (PPV HC-Pro) was always used. When several agrobacterium strains were used for infiltration, they were mixed in equal ratios. Purple spots around infiltration zones signifying anthocyanin synthesis were observed first on abaxial side of *N*. *tabacum* leaves starting from 3 dpi (days post infiltration). In case of very strong anthocyanin synthesis, a purple color was observed also on adaxial side of *N*. *tabacum* leaves in infiltration area. For *N*. *benthamiana*, reddish or orange spots were observed only on adaxial side of leaves. The photos were always taken at 7 dpi; *N*. *tabacum* was photographed from abaxial side; *N*. *benthamiana*—from adaxial side. The expression of infiltrated genes was verified by RT-PCR.

### Bioinformatics analysis

Manipulations of sequences was performed with Geneious software (Biomatters Ltd.), multiple sequence alignments made with ClustalW version 2.1, and phylogenetic trees created with MEGA version 6 [28] using the Neighbor-Joining method with 1,000 bootstrap repetitions. Sequences of *Prunus* anthocyanin pathway genes and cDNAs were obtained using BLAST search in NCBI NR and TSA databases, relative genes in *P*. *persica* and *P*. *mume* genomes were also identified by BLAST-search of NCBI WGS database and annotated manually.

RNA-Seq expression data for *P*. *avium* cultivar ‘Regina’ were obtained from a study where fruit samples were collected during 94 days weekly from the full bloom till the full ripeness (the first sample was the whole ovary tissue; all other samples were either exocarp, for all sampling points, or mesocarp, for two sampling points) [25]. Contigs resembling sequences of qPCR-analyzed genes were found, and expression data for all assembled fragments of that mRNA were analyzed. The RNA-Seq data were normalized according to the expression of the same actin gene, which was used in qPCR analysis. Raw sequences of *P*. *avium* RNA-Seq experiment were downloaded from NCBI SRA database.

## Results

### Isolation and characterization of anthocyanin pathway genes

At the start of this research, sequence information on some cherry anthocyanin synthesis genes as well as raw genome sequence of closely related peach species was already available in public databases. To add to this information, we isolated and sequenced full or partial UFGT, ANS, DFR, F3H, F3’H, CHI, CHS, PAL, WD40, bHLH3, bHLH33 and MYB10 cDNA sequences from sweet cherry cultivars ‘Irema BS’ and ‘Kitayanka’. In addition to cDNAs, we also cloned and sequenced genes or gene fragments of all abovementioned enzymes and regulators (except *PaF3H*, *PaF3’H* and *PabHLH33*) from ‘Irema BS’ and sour cherry cultivar ‘Balaton’. The gene for UFGT enzyme appeared to be extremely conserved with no nucleotide differences across all coding sequence and the only short intron in our sweet and sour cherries and another publicly available sweet cherry gene sequence (although several nucleotide differences are present among all available cDNA sequences). The strong homology persists also in available UFGT sequences of whole *Prunus* genus. Very homologous are also *PaANS*, *PaDFR* (the only available until now *P*. *avium* gene sequence clearly missed the second intron), *PaF3H* (no nucleotide differences in all known sweet cherry coding sequences), *PaF3’H*, *PaCHI*, *PaCHS* and *PaPAL* genes. At the amino acid level, all *Prunus* sp. flavonoid pathway enzymes share more than ninety percent of identical residues.

**Fig 2:**
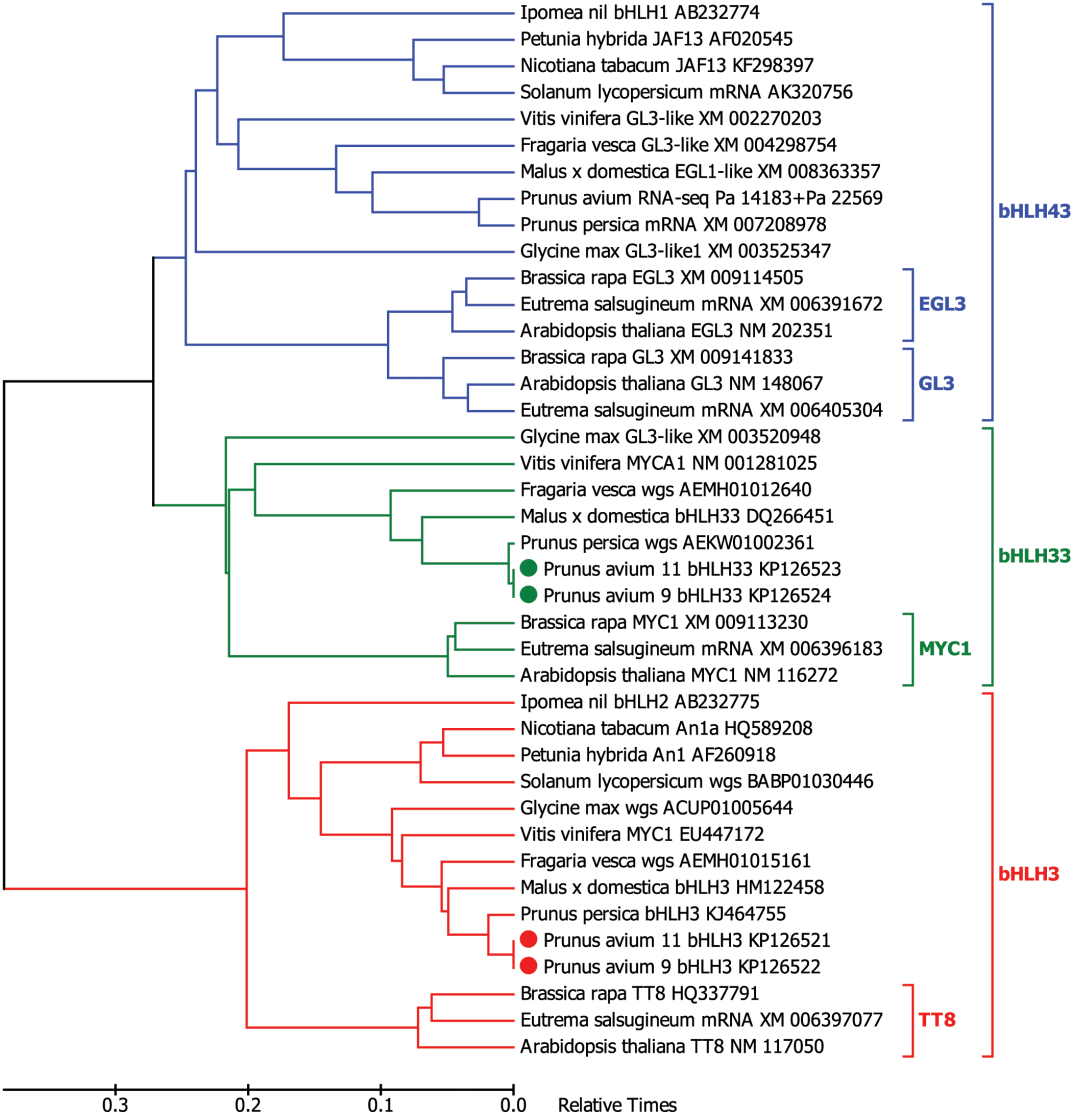
Phylogenetic timetree of related bHLH protein sequences from asterids and rosids clades. Divergence times for all branching points in the topology were calculated with the RelTime method using MEGA6. All proteins are named with species name, original protein name (as provided by a submitter), and accession number. Sources of unnamed sequences: RNA-Seq, sequences obtained from sweet cherry exocarp expression data; mRNA, translated unannotated cDNA sequences from nucleotide databases; wgs, protein sequences derived from genes found in the whole genome sequencing database. cDNAs for proteins which names are marked with full circles were isolated in this work; numbers 11 and 9 in these names mean cultivars ‘Irema BS’ and ‘Kitayanka’, respectively.

Among analyzed regulators of the anthocyanin pathway, the most conservative was intronless WD40 with identical amino acids and nucleotides across the whole *Prunus* genus reaching the level of enzymes and their coding sequences. The coding sequences and introns of bHLH genes, the largest among analyzed with complex structure containing eight introns, are also very conservative. Since homologous bHLH proteins were named differently in different plant species, we present here phylogenetic timetree of related bHLH protein sequences from asterids and rosids clades (Fig 2). As can be seen, two different bHLH genes encode proteins very similar to bHLH proteins named bHLH3 and bHLH33 in Rosaceae species, thus respective names were also given to the cloned *P*. *avium* genes.

### Several closely related MYB10 genes are present in the genomes of different *P*. *avium* cultivars

**Fig 3:**
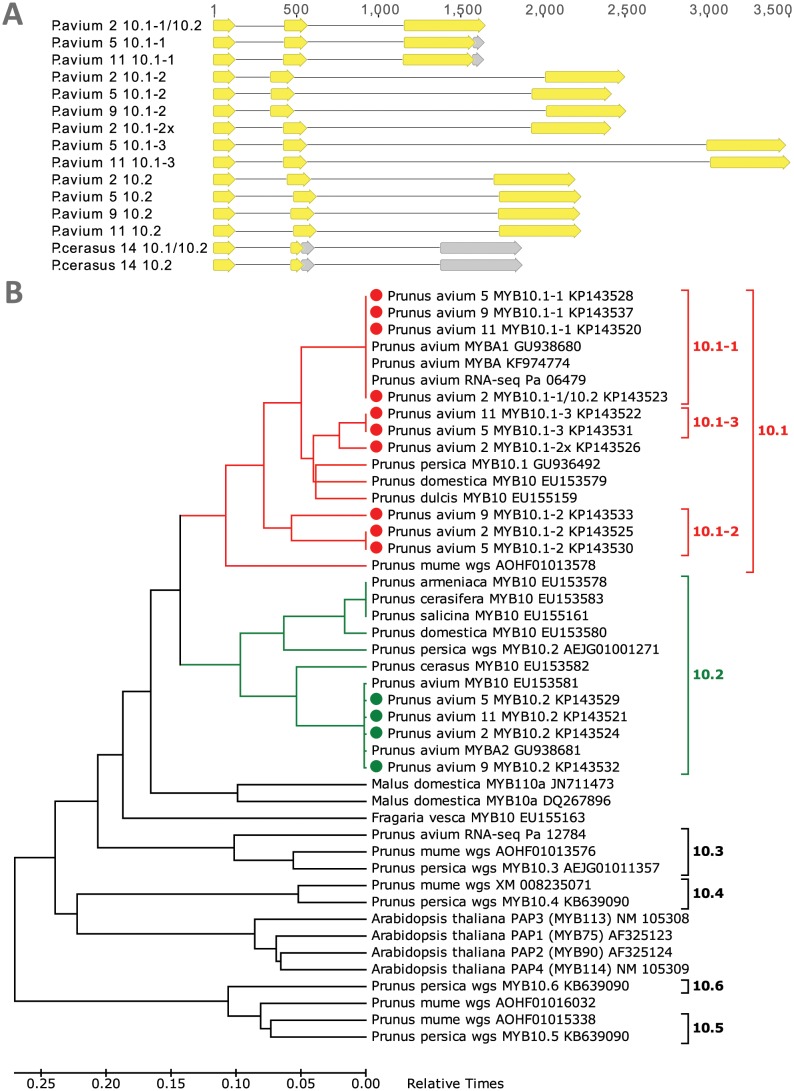
*Prunus avium* MYB10 genes. (A) Structures of *P*. *avium* and *Prunus cerasus* MYB10 genes cloned in this work. (B) Phylogenetic timetree of all *Prunus* MYB10 and related *Arabidopsis thaliana* protein sequences. The tree constructed and all proteins named exactly the same way as is described in the legend of Fig 2. Numbers 2, 5, 9, 11 and 14 denotes cultivars ‘Regina’, ‘Werdersche Braune’, ‘Kitayanka’, ‘Irema BS’, and ‘Balaton’, respectively.

Two different MYB10 variants were cloned from four *P*. *avium* cultivars: ‘Regina’, ‘Werdersche Braune’, ‘Kitayanka’ and ‘Irema BS’. By homology to the related peach genes, the shorter gene variant, about 1.6 kb in length, was named *PaMYB10*.*1*, and the longer variant, about 2.2 kb in length, was named *PaMYB10*.*2*. Both variants have very similar coding sequences, and the main differences reside in their introns. Putative proteins encoded by the *PaMYB10*.*1* variants are shorter by 21 amino acid (223 versus 244) because of the deletion of four nucleotides and resulting premature stop codon. Such gene variant from cultivar ‘Regina’ encodes full-length protein because of apparent recombination in the 3^rd^ exon and acquisition of the 3’-end from the variant MYB10.2. After PCR amplification of described genes, additional faint bands were visible slightly above MYB10.2 variant and a larger band, about 3.5 kb in length. After cloning and sequencing, it appeared that these bands contained another MYB10 variants, all coding for proteins more similar to MYB10.1 than to MYB10.2 variant, but without already mentioned deletion and premature stop codon (Fig 3A). Again, the main differences between new MYB10 variants were not in their coding sequences, but in introns. Since the coding sequences of these previously unknown variants were closely homologous to MYB10.1, the new subvariants were named *PaMYB10*.*1–2* (~2.5 kb), *PaMYB10*.*1-2x* (>2.5 kb, but encoded protein is more similar to *PaMYB10*.*1–3*) and *PaMYB10*.*1–3* (~3.5 kb). The ~1.6 kb variant, previously named as *PaMYB10*.*1*, was renamed to subvariant *PaMYB10*.*1–1*. Phylogenetic timetree of all known related MYB protein sequences from *Prunus* genus together with *Arabidopsis* anthocyanin-regulating MYBs and MYB10 proteins from apple and strawberry (Fig 3B) reveals that at least variants MYB10.1 and MYB10.2 are present also in other *Prunus* species. The remaining two anthocyanin-regulating *Arabidopsis* MYBs TT2 (MYB123) and MYBL2 were not included into this phylogenetic timetree, because they are more distant evolutionary.

### Expression of anthocyanin pathway genes in fruits of different *P*. *avium* cultivars

**Fig 4:**
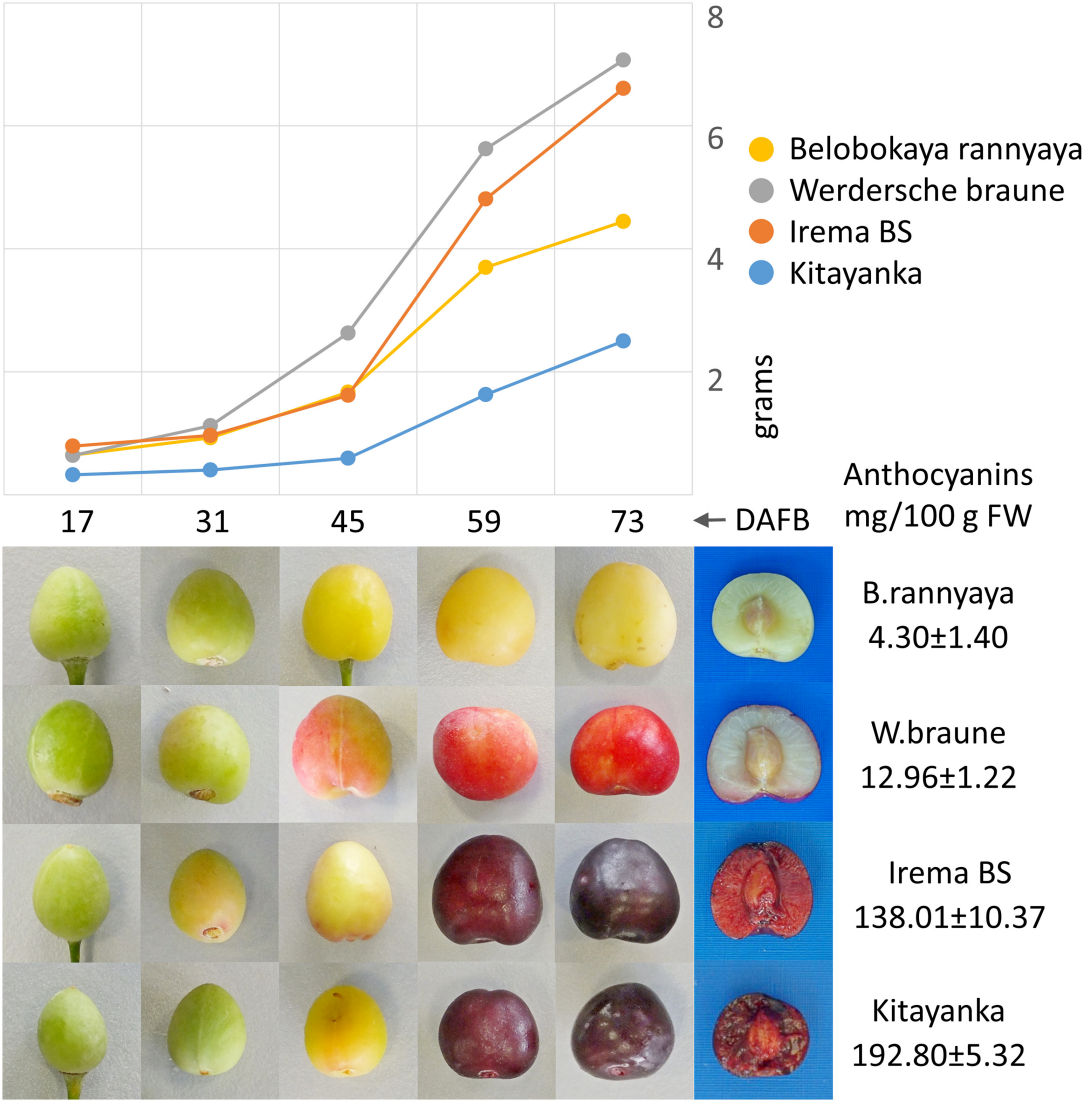
Analyzed sweet cherry fruit samples. (Top) Average fruit weight at five maturity stages denoted by DABF (days after full blossom). (Bottom) Fruits of each cultivar during development with cross-section and total anthocyanin content of mature fruits.

To determine how the expression of anthocyanin pathway genes and their regulators correlates with fruit color and anthocyanin production, we analyzed four cultivars with different levels of anthocyanins. Cultivar ‘Kitayanka’ produces relatively small fruits with highest anthocyanin content and darkest skin and flesh; cultivar ‘Irema BS’ produces larger fruits and also accumulates anthocyanins in skin and flesh, but at lower levels; cultivar ‘Werdersche braune’ accumulates anthocyanins only in the skin as a result of exposure to sunlight (so-called “blush” phenotype), and cultivar ‘Belobokaya rannyaya’ has yellow ripe fruits with some reddening of the skin during ripening (Fig 4). In addition, we analyzed data provided by the transcriptomics research of sweet cherry cultivar ‘Regina’ fruit exocarp development. Fruits of cultivar ‘Regina’ accumulate anthocyanins in both skin and flesh, thus are visually similar to fruits of cultivars ‘Kitayanka’ or ‘Irema BS’. According to our data, ‘Regina’ fruits produce less anthocyanins than ‘Kitayanka’, but more than ‘Irema BS’ fruits.

**Fig 5:**
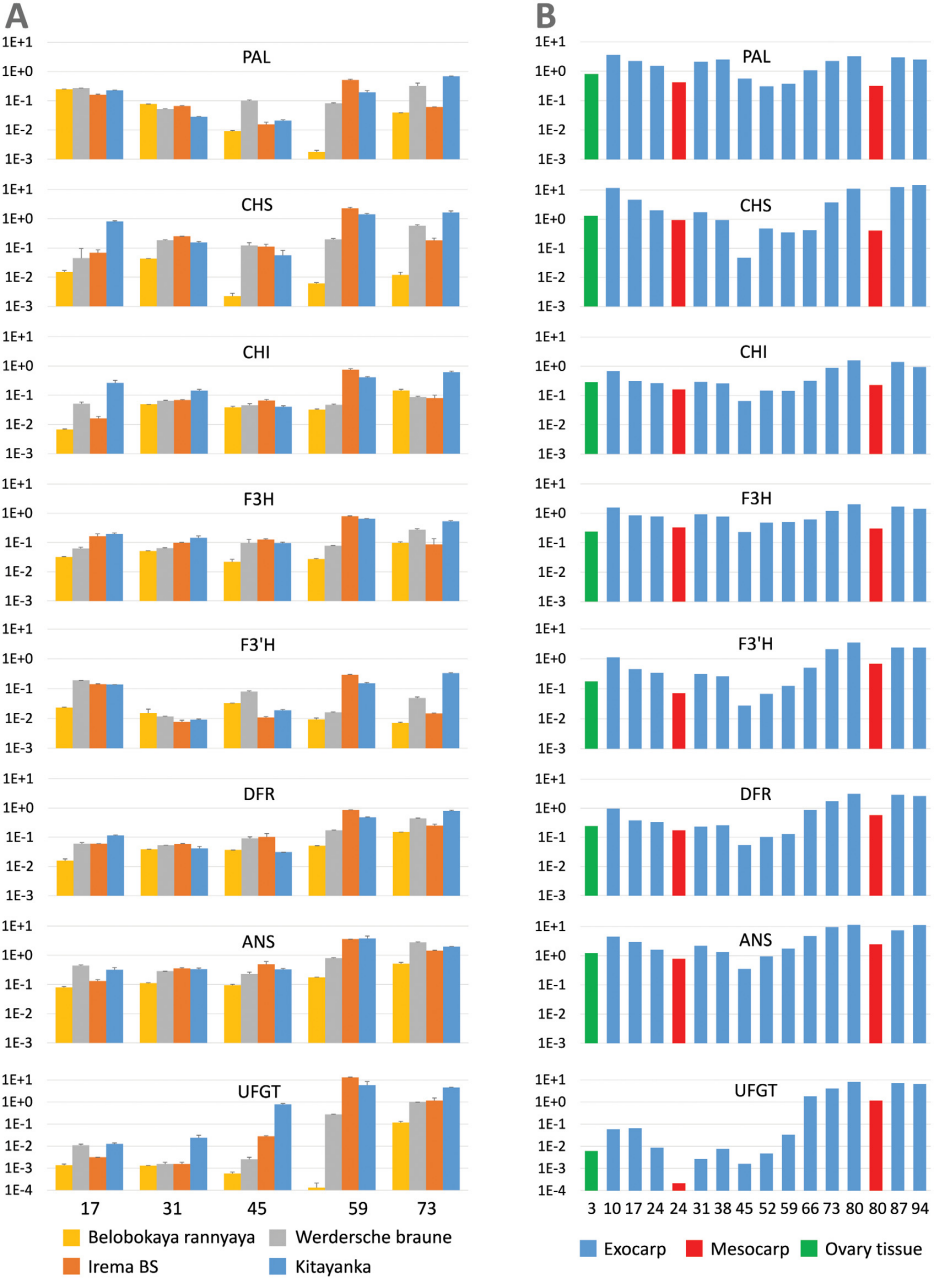
Expression of flavonoid pathway genes in fruits of different sweet cherry cultivars. (A) Real-time PCR expression data of different sweet cherry cultivars. (B) RNA-Seq expression data of fruit exocarp from sweet cherry ‘Regina’ cultivar. Only the data for the most abundant assembled fragment of each transcript is shown in RNA-Seq. All data are shown on a logarithmic scale and normalized by actin expression level.

According to qPCR results, the expression of genes participating in the late stages of anthocyanin synthesis was highest at the final stages of fruit development. The expression of most genes except *PaUFGT* and *PaANS* was lowest in the middle of the development in cultivar with highest anthocyanin level (Fig 5A). Comparison with RNA-Seq data revealed, that the most similar expression pattern for many genes was in fruits of cultivar ‘Kitayanka’ (Fig 5A). Overall normalized expression levels in qPCR analysis were lower than in RNA-Seq exocarp samples, but usually higher than in mesocarp samples because most of the material in our samples is represented by the mesocarp tissue. When comparing expression data of different cultivars, it is obvious that expression of most genes is highest in cultivar with highest anthocyanin production and lowest in cultivar with lowest anthocyanin levels, especially large difference is in the expression of *PaUFGT* and *PaCHS* of mature fruits. Although expression of most enzyme-coding genes in mature fruits was higher in cultivar with white-flesh fruits ‘Werdersche braune’ than in cultivar ‘Irema BS’, the higher anthocyanin content of ‘Irema BS’ fruits was probably determined at stage 4 (sampled at 59 DAFB), where expression of almost all enzyme-coding genes was even higher than in cultivar ‘Kitayanka’. The expression of all enzyme-coding genes is detectable in all analyzed cultivars, and this means that differences in anthocyanin production observed in different cultivars are probably determined not by the alterations in genes of individual enzymes, but in the expression level of regulators of the pathway.

### Expression of the regulatory genes

**Fig 6:**
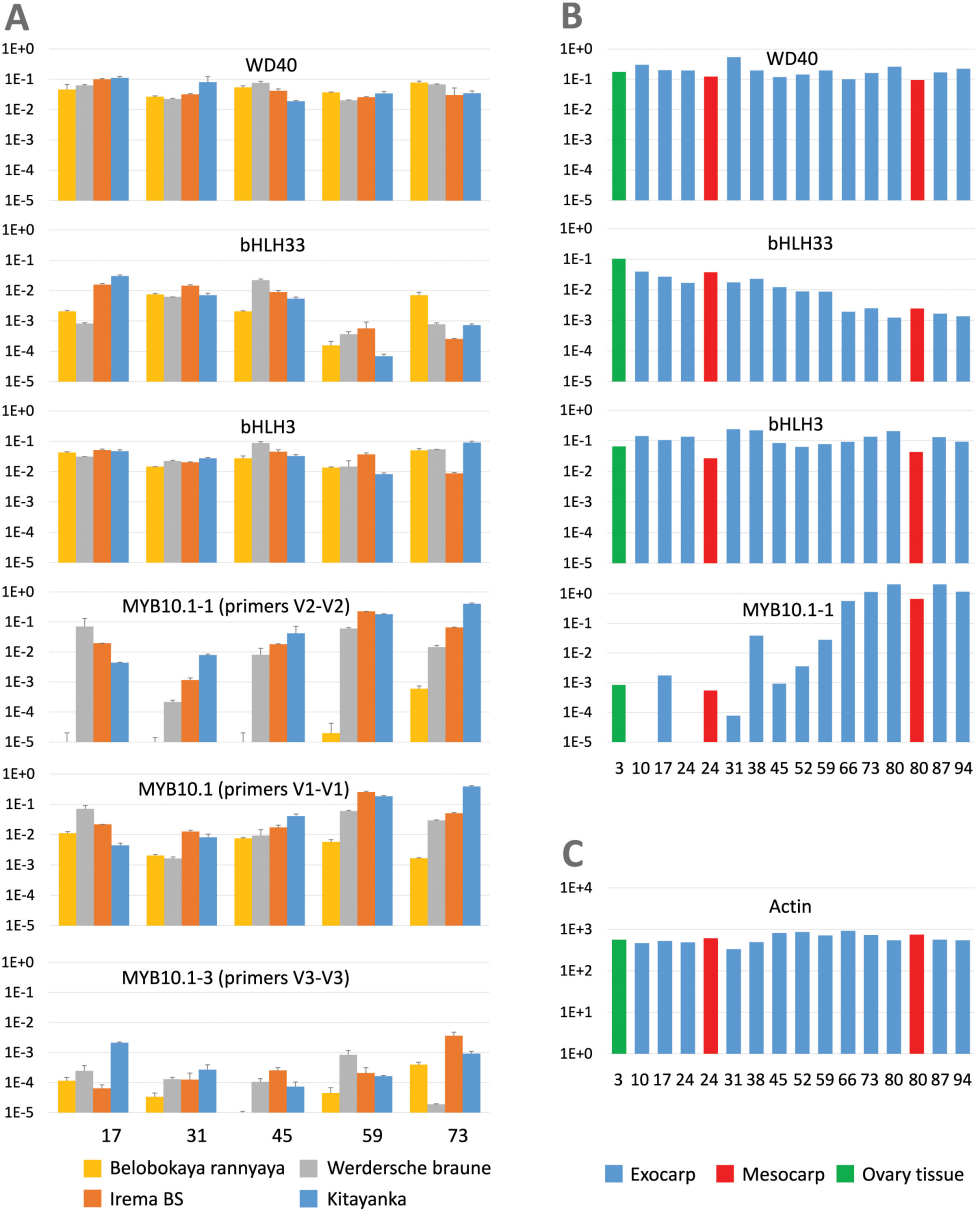
Expression of putative regulatory genes in fruits of different sweet cherry cultivars. (A) Real-time PCR expression data of different sweet cherry cultivars. V1-V1, V2-V2, and V3-V3 are different primer pairs used to analyze the expression of different MYB10 variants (explained in detail in text and in S1 Fig). (B) RNA-Seq expression data of fruit exocarp from sweet cherry ‘Regina’ cultivar. (C) RNA-Seq expression data for Actin. All data are presented as described in legend to Fig 4. (A) and (B)—data normalized by actin expression. (C)—absolute data in FKPM.

The analysis of putative regulators revealed that expression levels of *PaWD40* are relatively constant in all analyzed cultivars and at all maturation time points, and this was confirmed by the RNA-Seq data (Fig 6A). The expression patterns of two bHLH genes differed significantly. The expression of *PabHLH3* was relatively invariable and did not positively or negatively correlate with anthocyanin content in different cultivars and at different maturation stages. The same was true for RNA-Seq data, but there we also noticed a lowered expression of *PabHLH3* in the mesocarp tissue (Fig 6B). Meanwhile, the expression of *PabHLH33* varied significantly and inversely correlated with the expression of late anthocyanin biosynthesis genes at least in fruits with highest anthocyanin levels. The same pattern could be seen very clearly in RNA-Seq data, where *PabHLH33* expression gradually diminishes in samples from blossom towards mature fruit.

The expression analysis of previously isolated MYB10 gene variants showed, that variant *PaMYB10*.*1* was highly expressed in fruits with high anthocyanin levels, while the expression of variant *PaMYB10*.*2* was almost undetectable and not shown here. After the cloning of additional MYB10.1 variants and the division of this group into *PaMYB10*.*1–1*, *PaMYB10*.*1–2*, and *PaMYB10*.*1–3* subvariants, it appeared that primer pair MYB10 V1-V1 was capable of amplifying subvariant *PaMYB10*.*1–3* along with *PaMYB10*.*1–1*, thus we designed two new primer pairs, one specifically recognizing *PaMYB10*.*1–1* (primer pair MYB10 V2-V2) and one recognizing subvariants *PaMYB10*.*1–3* and *PaMYB10*.*1–2* (primer pair MYB10 V3-V3). Subvariant *PaMYB10*.*1–2* can be specifically analyzed also with primer pair MYB10 V0-V1 (details in S1 Fig). The expression analysis with additional primer pairs revealed that expression of *PaMYB10*.*1–1* (primer pair MYB10 V2-V2) was highest, whereas expression of *PaMYB10*.*1–3* (or *PaMYB10*.*1-2x*, primer pair MYB10 3–3) was detectable but low and not correlated with anthocyanin synthesis levels. The expression of *PaMYB10*.*1–2*, as revealed by the analysis with primer pair MYB10 V0-V1, was unnoticeable and data not shown. The analysis of RNA-Seq data produced the same results: the expression of *PaMYB10*.*1–1* was very high at the final stages of fruit development, whereas the expression of *PaMYB10*.*2* variant was almost undetectable. During analysis of sweet cherry RNA-seq data, we also found variant *PaMYB10*.*3*, which was not cloned in our work, but already analyzed in peach. The expression of *PaMYB10*.*3* variant in RNA-Seq data was lower than that of *PaMYB10*.*1–1*, higher at the end of fruit development than at the beginning (no expression at the middle) and was essentially absent in the mesocarp (data available in S1 File). The analysis of RNA-Seq data also revealed that control gene for the normalization of expression was chosen correctly, because it is expressed at high level and evenly in all developmental stages in exocarp, mesocarp and ovary tissue (Fig 6C).

### Transient expression of *P*. *avium* regulatory genes in leaves of Nicotiana plants

**Fig 7:**
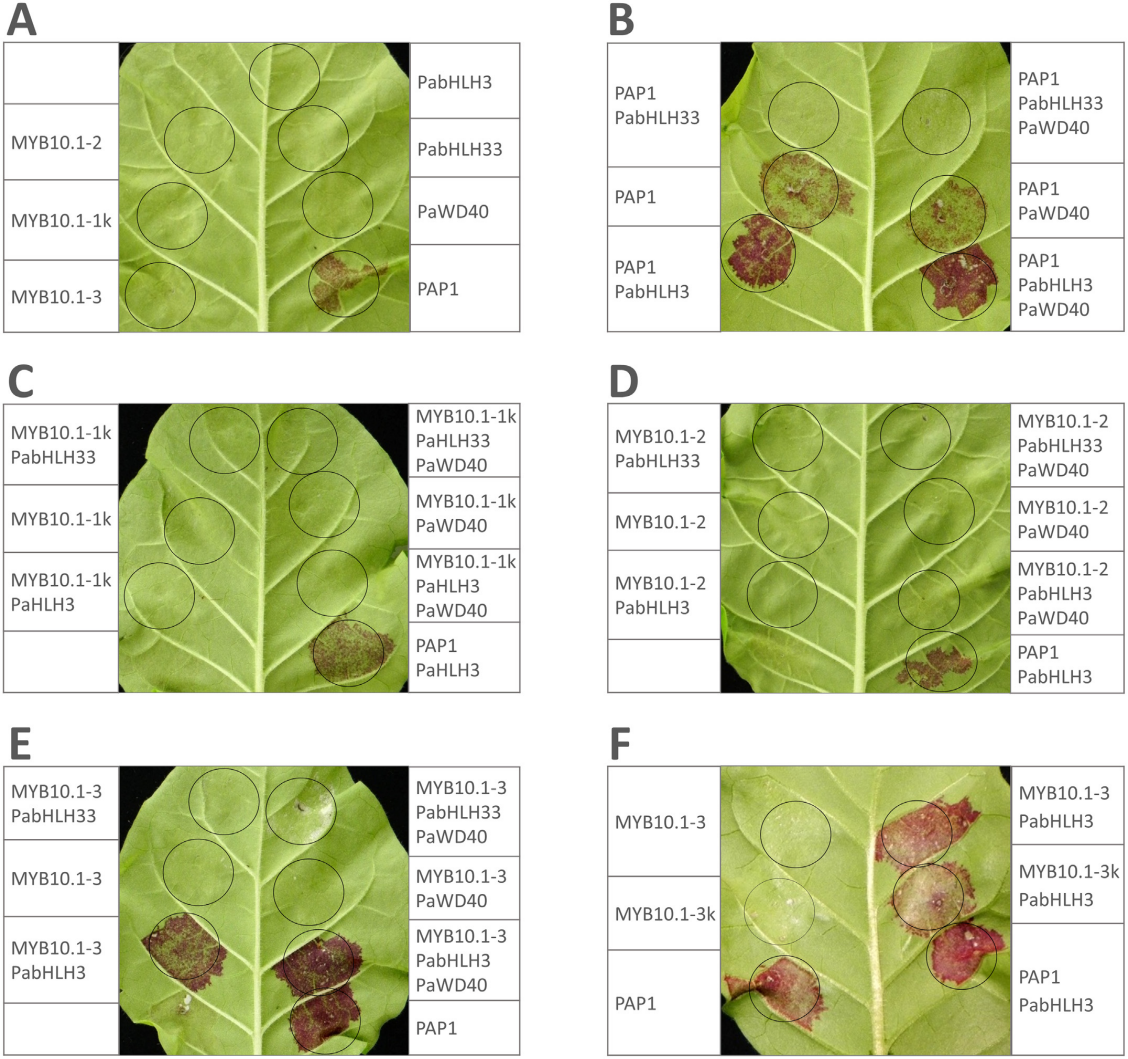
Transient expression of putative regulators of anthocyanin synthesis in leaves of *Nicotiana tabacum*. (A) Infiltrations of single genes. (B) The effect of *PaHLH3* and *PaHLH33*. (C) Infiltrations of *PaMYB10*.*1–1*. (D) Infiltrations of *PaMYB10*.*1–2*. (E) Infiltrations of *PaMYB10*.*1–3*. (E) A comparison between infiltrations of *PaMYB10*.*1–3* and *PaMYB10*.*1-3k*. Infiltrated control cDNAs: PAP1, *Arabidopsis thaliana PAP1* (MYB75); GFP, *Aequorea victoria GFP*. Tested *Prunus avium* genes are isolated from following cultivars: *PaWD40*, *PaHLH33*, *MYB10*.*1–1*, *MYB10*.*1–3* from ‘Irema BS’; *PaHLH3* from ‘Kitayanka’; *MYB10*.*1–3* from ‘Regina’. *MYB10*.*1-1k*—cDNA from ‘Kitayanka’ fruits; *MYB10*.*1-3k*—cDNA from infiltrated ‘Irema BS’ gene expressed in *N*. *benthamiana* leaves.

We tested all isolated cherry MYB10 gene variants alone and together with the *PabHLH3*, *PabHLH33* or *PaWD40* genes for the ability to induce anthocyanin synthesis in *N*. *tabacum* and *N*. *benthamiana* leaves. A positive control in these experiments was *A*. *thaliana PAP1* (MYB75), and a negative control was jellyfish *GFP* gene. A comparison of *N*. *tabacum* and *N*. *benthamiana* systems revealed that induction of anthocyanin synthesis in *N*. *tabacum* produces clear red patches on the bottom (abaxial) side of the leaf, whereas the second system produces red-yellow patches on the top (adaxial) side of the leaf and is generally more sensitive but more prone to overexpression-induced necrosis, especially when anthocyanin synthesis is high. We show here the results of *N*. *tabacum* infiltration (Fig 7), but the results with *N*. *benthamiana* and experiments including additional genes and their combinations can be found in S2 File.

At first, we tested which single genes were able to induce anthocyanin synthesis—only *PAP1* did (Fig 7A). Next, we tested the ability of cherry bHLH regulators to modulate anthocyanin induction. *PabHLH3* gene clearly enhanced anthocyanin synthesis when infiltrated together with *PAP1* in both systems (Fig 7B). The ability of *PabHLH3* to enhance anthocyanin synthesis was later confirmed with infiltrations together with *PaMYB10*.*1–3* gene (Fig 7E and 7F). *PabHLH33* gene, on the contrary, completely inhibited anthocyanin synthesis induced by *PAP1* gene (Fig 7B). *PabHLH33* gene was also able to prevent anthocyanin induction when infiltrated together with *PaMYB10*.*1–3* and *PabHLH3*, also when infiltrated with another positive control SlANT1* (see S2 File). *PabHLH33* also prevented anthocyanin synthesis induced by *Ribes rubrum RrMYB10* gene (unpublished data). We also tested *PaWD40* in various combinations with other supposed members of MBW complex, but the addition of this gene had no distinctive effect on anthocyanin synthesis (Fig 7B–7E).

Whereas all tested control and bHLH coding sequences were in the form of intronless cDNA, all tested MYB10 (except *PaMYB10*.*1–1*, which was available also in cDNA form) were cloned genome sequences. *PaMYB10*.*1–1*, which is highly expressed in anthocyanin-producing fruits, showed no effect on anthocyanin induction either alone or together with any bHLH and *PaWD40* genes (Fig 7C). The same results were obtained with the intron-containing gene sequence of the same MYB variant isolated from another cultivar (S2 File). *PaMYB10*.*1–2* was also unable to induce anthocyanin synthesis in any combination in both *Nicotiana* species (Fig 7D). The only MYB variant able to induce anthocyanins was *PaMYB10*.*1–3*, which is low-expressed in fruits (Fig 7E). In *N*. *benthamiana* system, *PaMYB10*.*1–3* induced anthocyanin synthesis alone (shown in S2 File), whereas the addition of *PabHLH3* was needed in *N*. *tabacum* infiltrations. Again, the addition of *PabHLH33* inhibited all anthocyanin production (S2 File). Since MYB10.1–3 gene has very long introns, especially the second, we tested the possibility that these noncoding sequences determine or contribute to the induction of anthocyanin synthesis. Thus, we isolated and sequenced *PaMYB10*.*1–3* cDNA from infiltrated *N*. *benthamiana* plants and found that RNA was processed as expected and encoded predicted MYB protein. The infiltration of *PaMYB10*.*1–3* cDNA revealed that it was also able to induce anthocyanin synthesis, although at slightly lower level (Fig 7F). Again, the addition of *PaWD40* gene had no effect on anthocyanin synthesis. We infiltrated also *PaMYB10*.*1–3* gene isolated from different cultivar, and it also induced anthocyanin synthesis in tobacco when applied together with *PabHLH3* (S2 File).

## Discussion

### The role of different bHLH proteins in the regulation of anthocyanin biosynthesis

In this study, we isolated and sequenced whole or partial genes or cDNAs encoding anthocyanin pathway enzymes or regulatory proteins from several sweet cherry cultivars. In general, the sequence analysis confirmed a high degree of conservation of all enzymes and regulators bHLH and WD40 across the whole *Prunus* genus. Almost all of these genes have clearly identifiable counterparts in species from asterids and rosids clades, for which relevant sequence information is available. This probably means that homologous genes have identical or very similar functions in different organisms, and such assumption is constantly confirmed by functional analysis and phenotypes of transgenic plants [29]. Although early anthocyanin pathway research was done in flowers such as snapdragon [30] and petunia [31], functions of different plant proteins are usually inferred by their homology to proteins of model species *A*. *thaliana*, and inferred function suggests further experiments and their interpretation. In this aspect, some uncertainty persisted concerning anthocyanin-related bHLH proteins. Despite clear homology of analyzed bHLH sequences to their bHLH3 and bHLH33 counterparts in other Rosaceae species, there was some confusion to which proteins of *A*. *thaliana* they are related. The main three bHLH family proteins involved in anthocyanin regulation are TT8, EGL3 and GL3. According to current understanding, all these proteins are participating in MBW complex at different stages of development or in different tissues, and their overexpression usually upregulates anthocyanin synthesis or restores its deficiency. In the case of bHLH3, there is little doubt that this protein is the homolog of *Arabidopsis* TT8, and experiments showed that apple bHLH3 can assist MYB10 in activating *Arabidopsis* DFR promoter in tobacco [18, 20] and promotes anthocyanin accumulation in response to low temperatures in apples [27]. Our expression analysis shows that bHLH3 is stably expressed in fruits, and transient expression experiments confirm it as a strong positive modulator of anthocyanin induction when applied together with some MYB proteins.

At the same time, the relatedness of bHLH33 to any *Arabidopsis* anthocyanin-regulating bHLH remained unclear, and several similar proteins in other species were named GL3-like. However, phylogenetic tree clearly shows that bHLH33 is more related to *Arabidopsis* MYC1 protein, and that similar proteins are present only in rosids, but not asterids clade (Fig 2). Whereas EGL3 and GL3 are known as positive regulators of anthocyanin synthesis, the ability of *Arabidopsis* MYC1 to regulate anthocyanin synthesis is unclear. Our qPCR results and the analysis of RNA-Seq data reveals that the expression of bHLH33 gradually diminishes during maturation in cultivars with highest anthocyanin content. The declining of bHLH33 expression is especially obvious in RNA-Seq data. It must be emphasized, that all data in diagrams are presented in logarithmic scale to embrace the high dynamic range of expression differences and are normalized to actin. Essentially, the RNA-Seq expression data in the range of 10^–3^ means that there are only few reads per million aligning to the kilobase of analyzed RNA. (All raw qPCR and RNA-Seq data, and their diagrams with linear and logarithmic scales, as well as RNA-Seq data for additional genes of the flavonoid pathway are available in S1 File) Similarly, the lowered expression of bHLH33 in anthocyanin-rich parts of fruit is also noticeable in peach [10]. Although published functional tests showed some ability of apple bHLH33 to activate *Arabidopsis* DFR promoter together with MYB10 in tobacco, its ability to assist in inducing anthocyanin synthesis was not shown [18,20]. Our transient expression results do not confirm the possibility that sweet cherry bHLH33 facilitates anthocyanin induction and even defines it as a negative modulator. The ability to suppress anthocyanin synthesis in infiltration experiments is interesting, but it remains to establish whether this phenomenon is pertinent specifically to cherry (or *Prunus*) bHLH33 and whether it is specific to anthocyanin synthesis genes.

The phylogenetic tree (Fig 2) also reveals, that another bHLH protein, found in asterids as well as in rosids, is distant relative of *Arabidopsis* EGL3 and GL3. Thus, another likely positive regulator of anthocyanin synthesis might be the protein belonging to a branch labeled here as bHLH43. Although we found relevant coding sequences in genomics and transcriptomics databases, no one protein of this branch belonging to Rosaceae family species has been investigated until now.

### The abundance of sweet cherry MYB10 gene variants reveals complexity and ongoing evolution of anthocyanin synthesis regulation

The sequences of two sweet cherry MYB10 gene variants were known at the beginning of this research. We isolated and sequenced several additional subvariants from different cultivars with coding sequences very similar to one of the variants. Three similar MYB10 gene variants named *PpMYB10*.*1*, *PpMYB10*.*2* and *PpMYB10*.*3* were found in peach genome [10]. Two sweet cherry variants isolated earlier are clear homologs of the peach MYB10.1 and MYB10.2, and we named them accordingly. We have not isolated *P*. *avium* homolog of MYB10.3 variant, but found it in available RNA-seq data. We also searched *P*. *persica* and *P*. *mume* genomes for sweet cherry counterparts of MYB10.1 subvariants, but instead found only more distantly related variants (Fig 3B). Recently, the latter gene variants, which are located on another genome area, different from the one where variants MYB10.1-MYB10.3 reside, were analyzed and named *PpMYB10*.*4*, *PpMYB10*.*5* and *PpMYB10*.*5* [12]. The expression of *PpMYB10*.*4* is clearly enhanced in red leaves, whereas functions of two remaining variants are unknown. It was proposed that the ancestral *MYB* gene have undergone a duplication ~ 70 million years ago and generated two gene families MYBI and MYBII. It is possible that sweet cherry subvariants *PaMYB10*.*1–1*, *PaMYB10*.*1–2* and *PaMYB10*.*1–3* are the result of another, more recent duplication. Unfortunately, we cannot prove this while genome sequence of *P*. *avium* is unavailable. Such variety of *Prunus* MYB10 genes suggests rapid ongoing evolution, but it is unclear which of them are active and how their functionalities differ. Although there is some data about different functions of closely related anthocyanin-regulating proteins in *Arabidopsis*, it is impossible to pair them with *Prunus* counterparts. Several similar MYB10 genes were found also in apple, some of them apparently control anthocyanin accumulation predominantly in fruit skin or flesh [32, 33]. The same distribution of functions can be suggested for different *Prunus* MYB10 variants, but direct comparison to apple proteins is impossible, because all known apple MYB10 proteins are closely homologous to each other and more distant to MYB10.1 and MYB10.2 variants, which probably originated already in *Prunus*. Different sweet cherry MYB10 variants can also regulate anthocyanin synthesis in other organs, for example in red leaves. In peach, this function is recently assigned to *PpMYB10*.*4* [12], which belongs to MYBII family. It is also possible that some of the newly discovered MYB10 variants can regulate anthocyanin synthesis in petals. However, the peach MYB regulator of petal coloration, named *Peace* [34], is more similar to *Arabidopsis* TT2 (MYB123) than to any MYB10-like protein.

The prime purpose in analysis of MYB10 expression was to clarify which of the variants (here named *PaMYB10*.*1* and *PaMYB10*.*2*) is highly expressed in red fruits, because two earlier studies [13, 21] analyzed expression of each variant separately with primer pairs capable of at least partially amplify the sequences of both variants. Our experiments, as well as the analysis of RNA-Seq data, confirmed that recently analyzed [13] variant MYB10.1, and namely subvariant *PaMYB10*.*1–1*, is expressed at high levels in fruits with higher anthocyanin content, whereas the expression of variant *PaMYB10*.*2* is almost undetectable. Among other analyzed MYB10.1 subvariants, only *PaMYB10*.*1–3* was expressed at low levels in fruits. The results of expression analysis also suggest the involvement of at least one additional MYB10 player. Primer pair V1-V1 designed to analyze MYB10.1 variants generates low, but clear signal in all maturation stages of cultivar ‘Belobokaya rannyaya’ fruits (Fig 6A). Meanwhile, primer pair V2-V2, destined to analyze specifically the expression of subvariant *PaMYB10*.*1–1*, shows its expression only in stage 5 fruits. The expression of *PaMYB10*.*1–3* cannot explain the signal generated by V1-V1, because the signal generated by primer pair V3-V3 is much lower. It can be supposed, that primer pair V1-V1 is capable to detect also expression of variant MYB10.3 (see S1 Fig), which is expressed at low level in anthocyanin-producing peach [10] and sweet cherry (S1 File) fruit tissues. However, even in ‘Belobokaya rannyaya’ fruits, the expression of at least *PaUFGT* gene is clearly correlated only with the expression of variant *PaMYB10*.*1–1* and once more confirms this MYB variant as the regulator of at least late anthocyanin-producing enzyme genes. Meanwhile, functions of low-expressed variants in anthocyanin regulation of sweet cherry and peach fruits remains unclear.

In transient expression experiments, *PaMYB10*.*1–1* was unable to induce anthocyanin synthesis, whereas *PaMYB10*.*1–3* did this with the help of *PabHLH3*. Since gene and cDNA sequences had very similar effect on anthocyanin induction, the differences in encoded protein sequences are probably responsible for the differences in functionality between different MYB10 subvariants. Why MYB10 variant predominantly expressed in cherry fruits is not the same that induces anthocyanin synthesis in tobacco? It is obvious that expression patterns and structures of anthocyanin regulators, as well as promoter sequences, in tobacco leaves differ at least to some degree from their counterparts in sweet cherry fruits. Some proteins of one species even do not have counterparts in another (for example, according to our analysis, there is no bHLH33 counterpart in tobacco). And there may be also other levels of regulation that differ between species and tissues. Thus, to answer this question, a detailed analysis of interactions between different MYB variants and proteins, as well as promoters, from tobacco and sweet cherry may be needed. And the similarity of protein sequences in different MYB10 variants provides the opportunity to readily pinpoint the exact protein regions responsible for any established functional differences.

## Supporting Information

S1 Fig Scheme of qPCR primers for different sweet cherry MYB10 gene variants. (PDF)

S1 File Microsoft Excel file with qPCR data and analyzed expression data for relevant genes from published RNA-seq study. (XLSX)

S2 File Additional experiments of transient expression in *Nicotiana tabacum* and *N*. *benthamiana* leaves. (PDF)

S1 Table Primers for gene cloning and cDNA synthesis. (PDF)

S2 Table Real-time PCR primers. (PDF)

S3 Table Primers used for the amplification of genes analyzed in transient expression assays. (PDF)
