# Supplementary material for: Expression and Anthocyanin Biosynthesis-Modulating Potential of Sweet Cherry (Prunus avium L.) MYB10 and bHLH Genes
Source: PLoS One. 2015 May 15;10(5):e0126991. doi: 10.1371/journal.pone.0126991 (PMC4433224; doi:10.1371/journal.pone.0126991)

Primers for qPCR of PaMYB10 gene variants

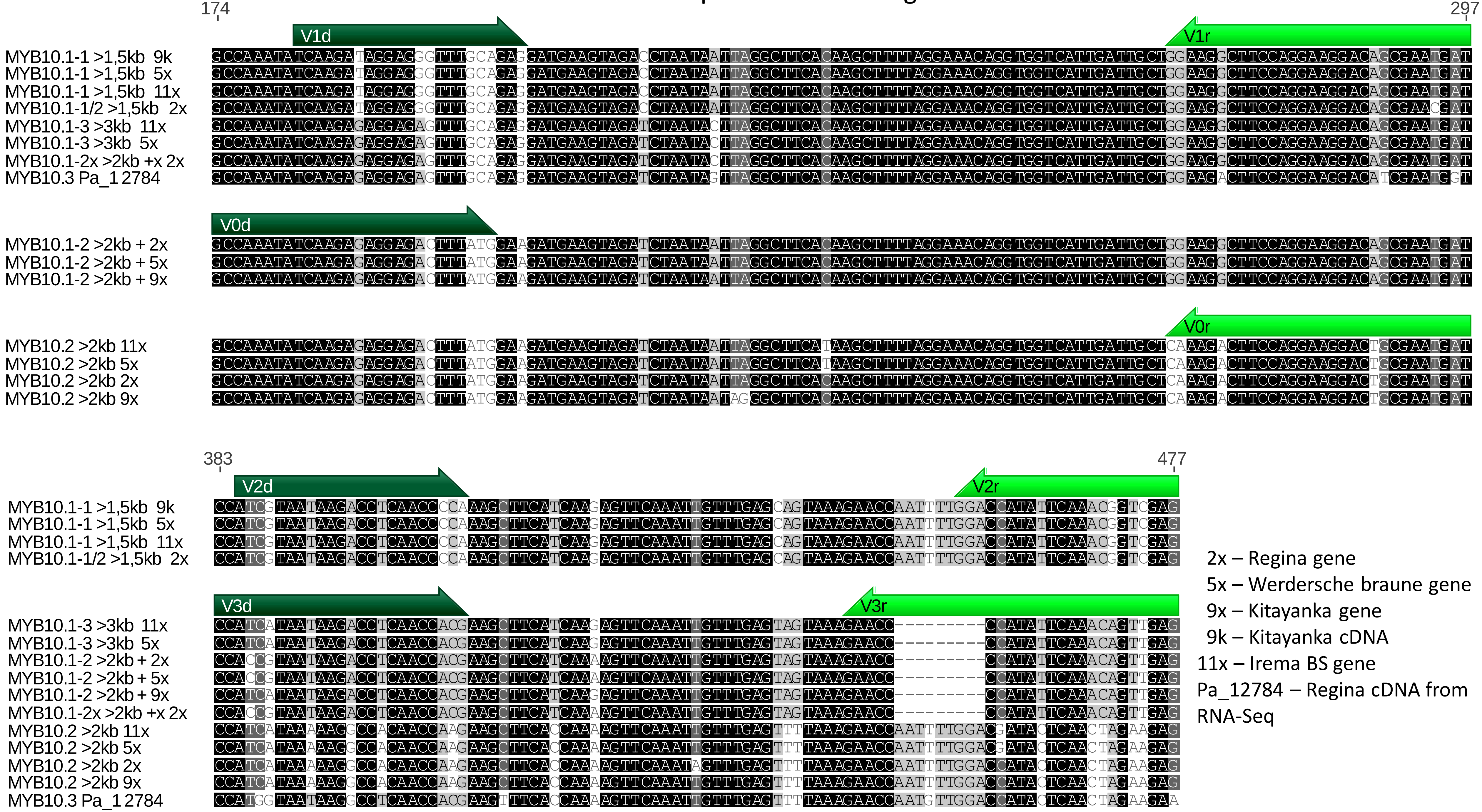

Supplement: S1 Fig — (PDF) [file pone.0126991.s001.pdf]
