# Supplementary material for: Expression and Anthocyanin Biosynthesis-Modulating Potential of Sweet Cherry (Prunus avium L.) MYB10 and bHLH Genes
Source: PLoS One. 2015 May 15;10(5):e0126991. doi: 10.1371/journal.pone.0126991 (PMC4433224; doi:10.1371/journal.pone.0126991)
Supplement: S2 File — (PDF) [file pone.0126991.s003.pdf]

Gene abbreviations

Abbreviation	Gene/cDNA	Species	Cultivar
PAP1	cDNA	Arabidopsis thaliana	
ANT1*	cDNA	Solanum lycopersicum	mutant D72N
GFP	cDNA	Aequorea victoria	
PaWD40	gene	Prunus avium	Irema BS
PaHLH3	cDNA	Prunus avium	Kitayanka
PaHLH33	cDNA	Prunus avium	Irema BS
MYB10.1-1k	cDNA	Prunus avium	Kitayanka
MYB10.1-1	gene	Prunus avium	Irema BS
MYB10.1-2	gene	Prunus avium	Regina
MYB10.1-3	gene	Prunus avium	Irema BS
MYB10.1-3k	cDNA	Prunus avium	Irema BS gene->N.benthamiana cDNA
MYB10.1-3a	gene	Prunus avium	Werdersche braune

Table of infiltrated genes – on the top or on the side of the leaf (leaves).
In the left column - infiltrated genes on the left side of a single leaf.
In the right column - infiltrated genes on the right side of a single leaf.
Leaves bordered without gaps are infiltrated exactly the same and share the same table.

Single genes

GFP	PaHLH3
MYB10.1-2	PaHLH33
MYB10.1-1k	PaWD40
MYB10.1-3	PAP1

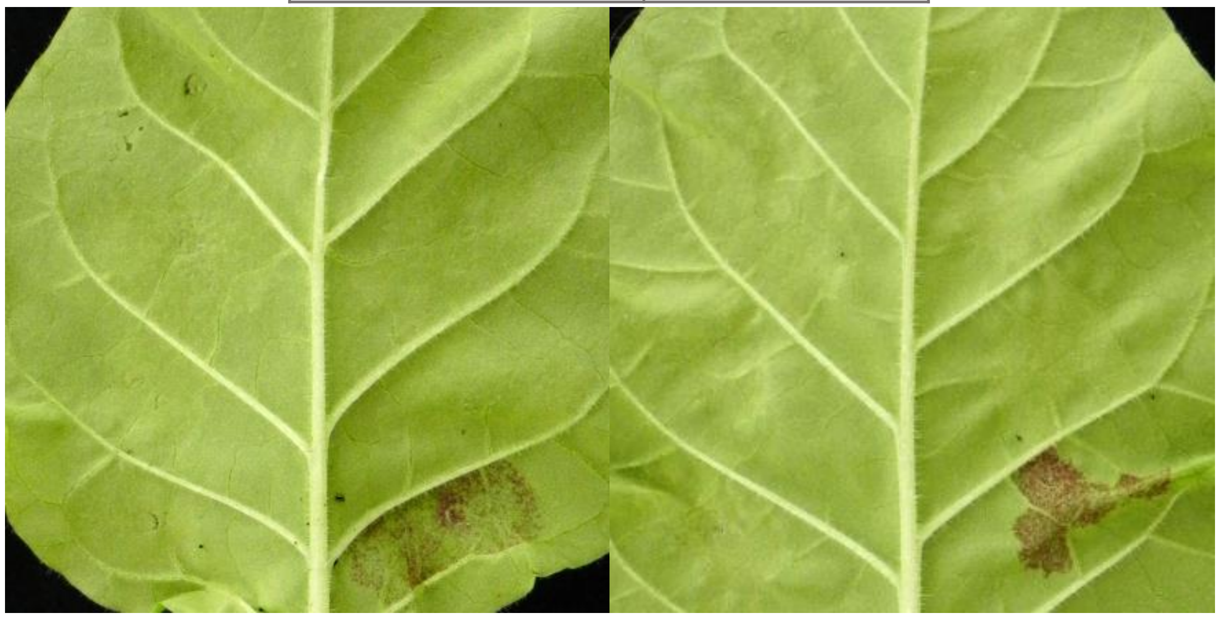

ANT1*	PaHLH3
GFP	PaHLH33
PAP1	MYB10.1-3

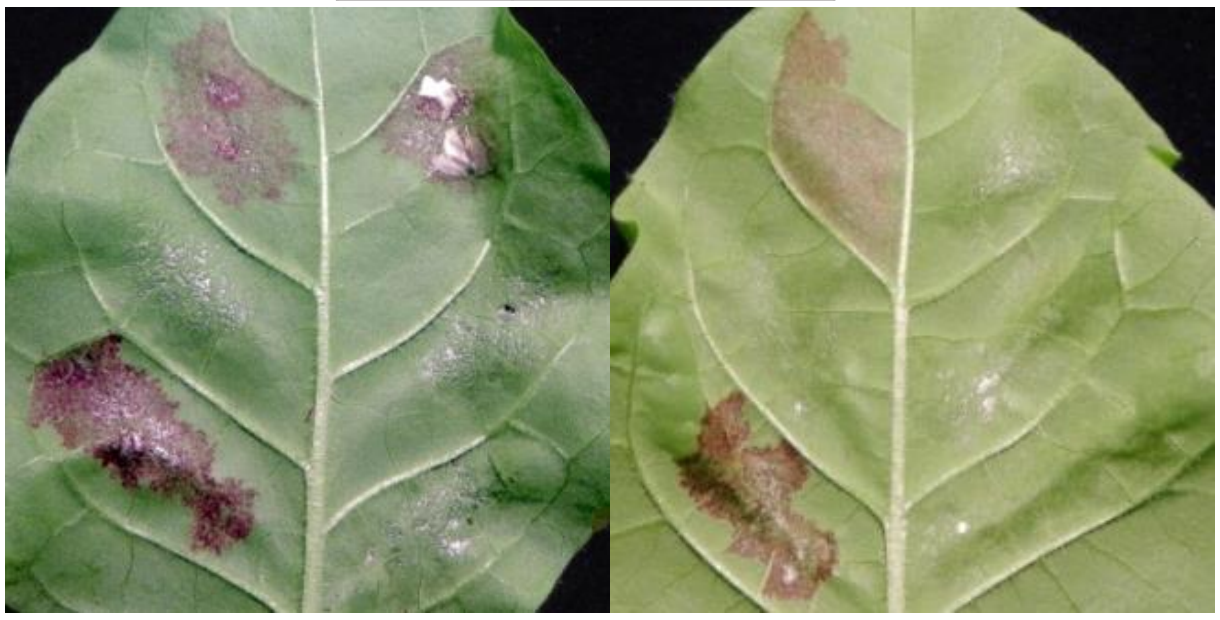

PAP1	GFP
GFP	MYB10.1-1k
ANT1*	MYB10.1-2

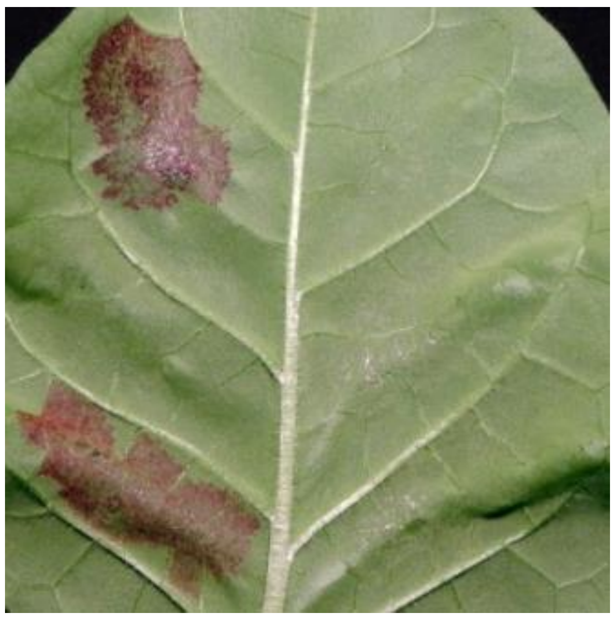

Nicotiana tabacum

GFP	MYB10.1-2
	MYB10.1-1k
ANT1*	MYB10.1-3

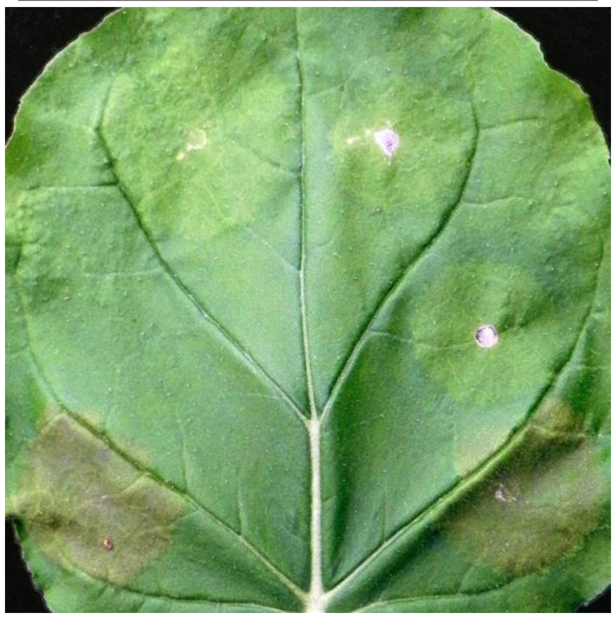

PAP1	GFP
GFP	MYB10.1-1k
ANT1*	MYB10.1-2

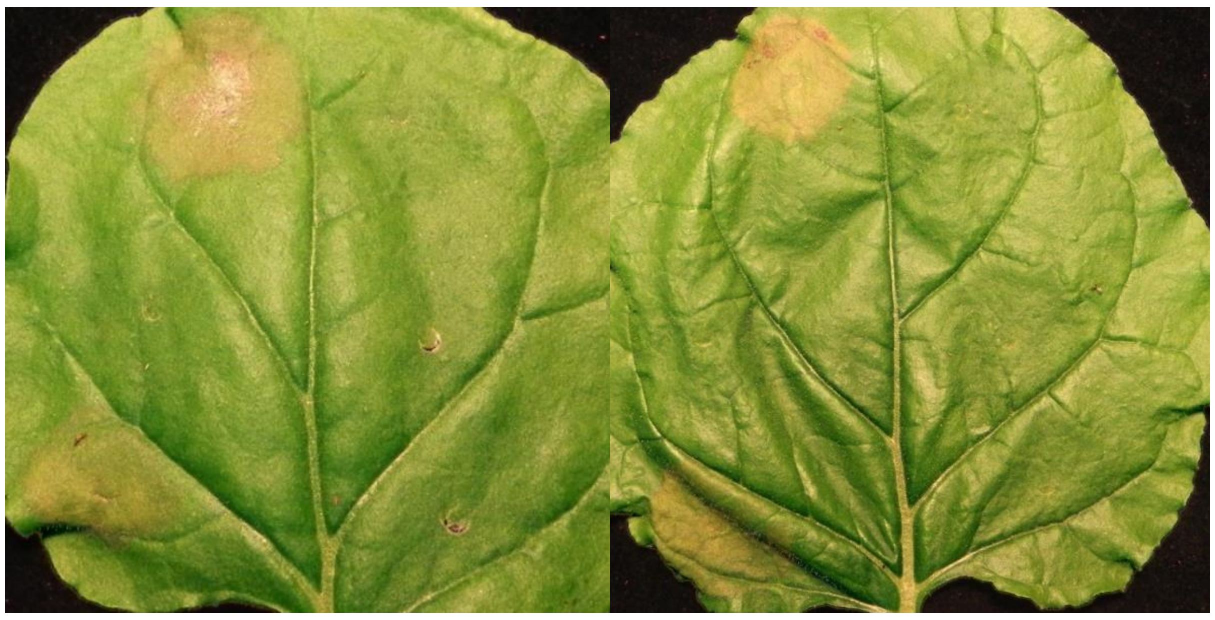

ANT1*+GFP	ANT1*
GFP+GFP	GFP
PAP1+GFP	PAP1

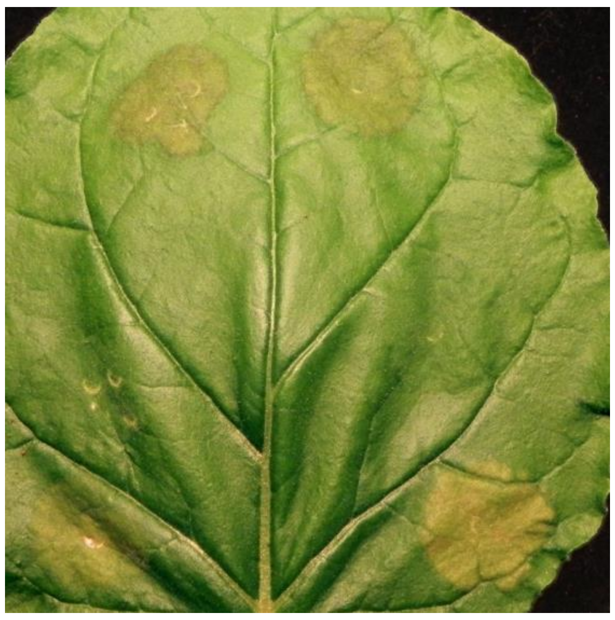

Nicotiana benthamiana

PaHLH3 - PaHLH33

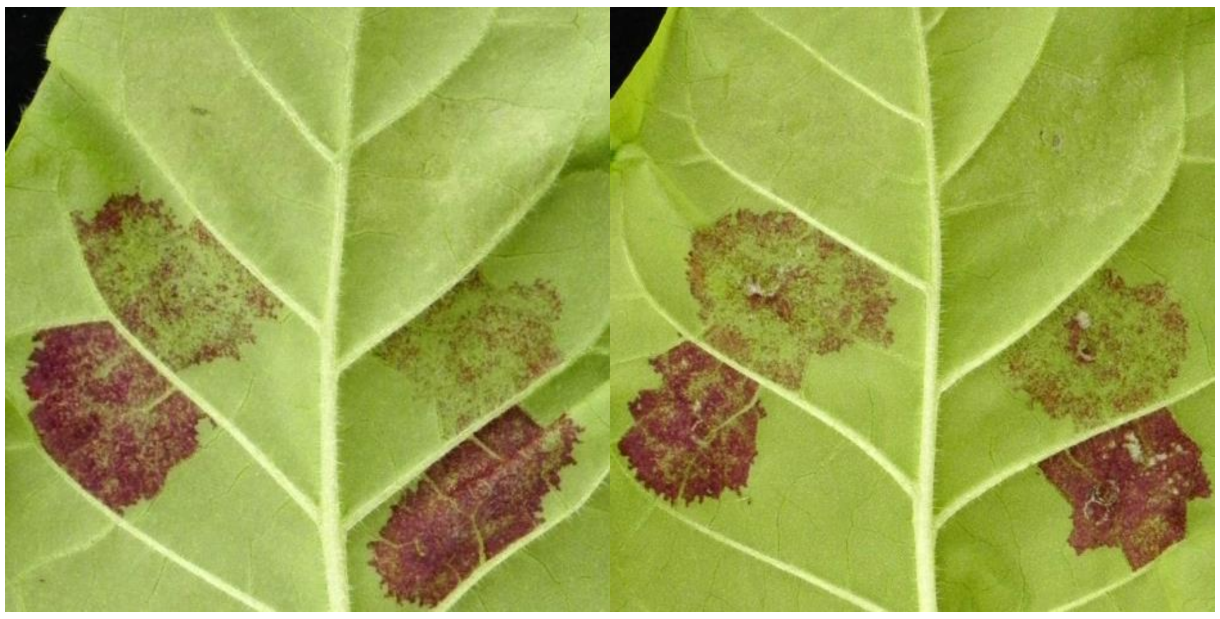

PAP1+PaHLH33	PAP1+PaHLH33+PaWD40
PAP1	PAP1+PaWD40
PAP1+PaHLH3	PAP1+PaHLH3+PaWD40

PAP1+PaHLH33+PaWD40	PAP1+PaHLH33
PAP1+PaWD40	PAP1
PAP1+PaHLH3+PaWD40	PAP1+PaHLH3

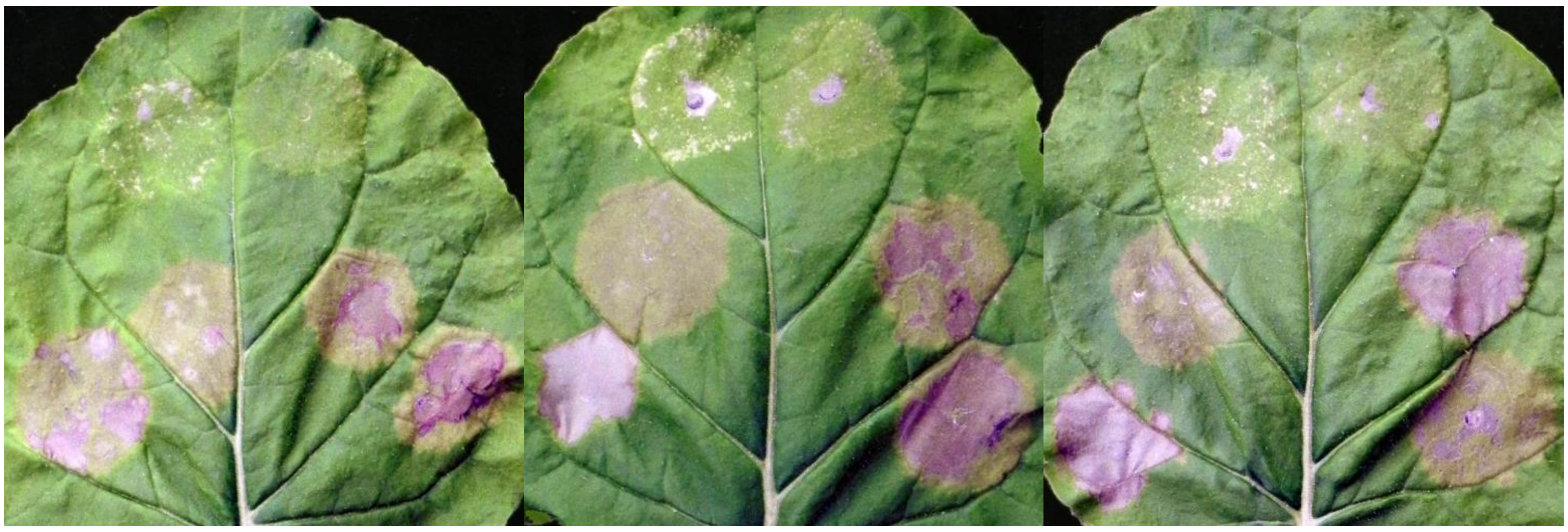

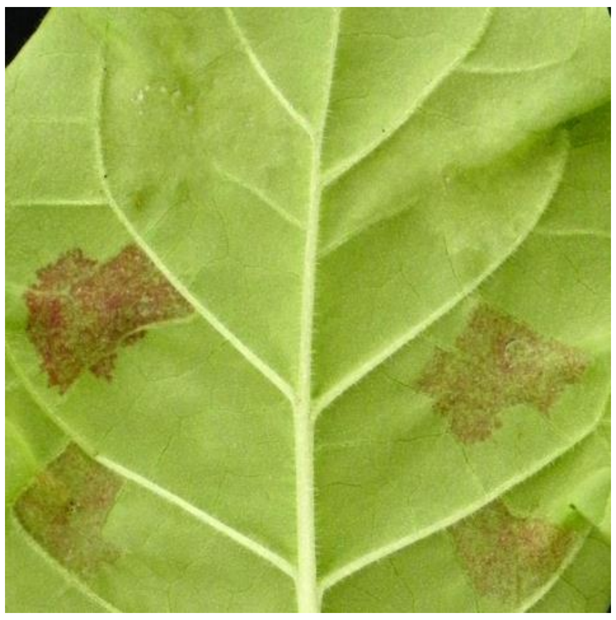

ANT1*+PaHLH33	ANT1*+PaHLH33+PaWD40
ANT1*+PaHLH3	ANT1*+PaHLH3+PaWD40
ANT1*	ANT1*+PaWD40

ANT1*+PaHLH33+PaWD40	ANT1*+PaHLH33
ANT1*+PaWD40	ANT1*
ANT1*+PaHLH3+PaWD40	ANT1*+PaHLH3

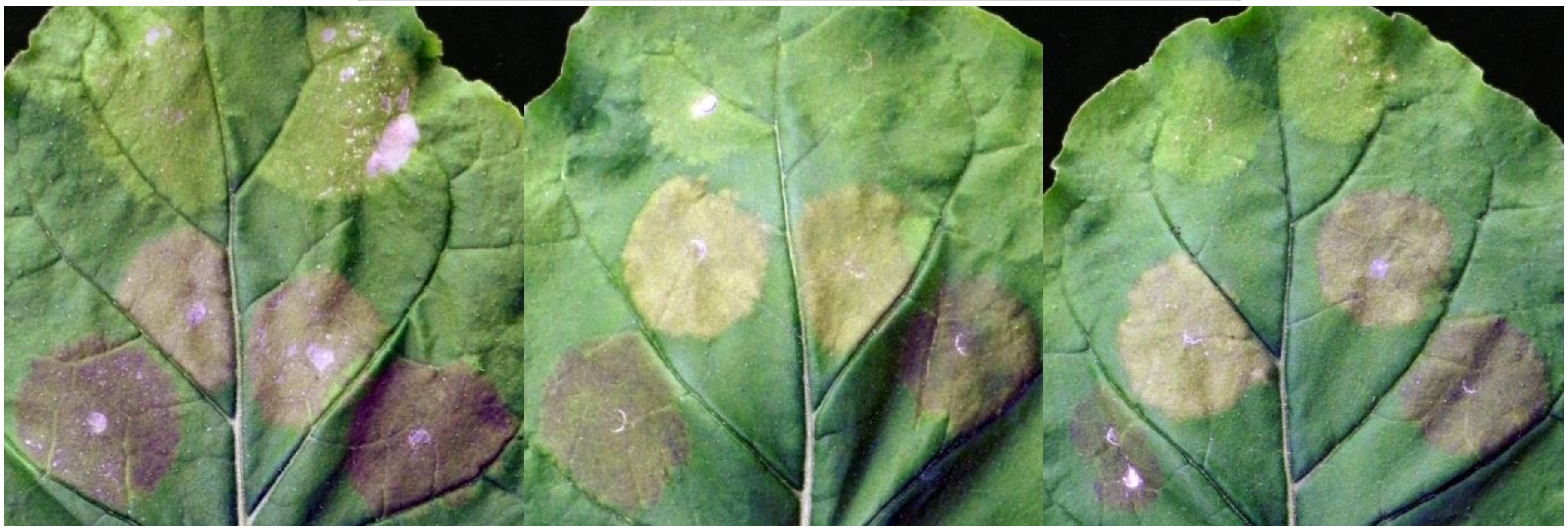

Nicotiana tabacum

Nicotiana benthamiana

PaMYB10.1-1k - PaMYB10.1-1

MYB10.1-1k	MYB10.1-1k+PaHLH3
GFP	MYB10.1-1k+GFP
PAP1	MYB10.1-1k+PaHLH33

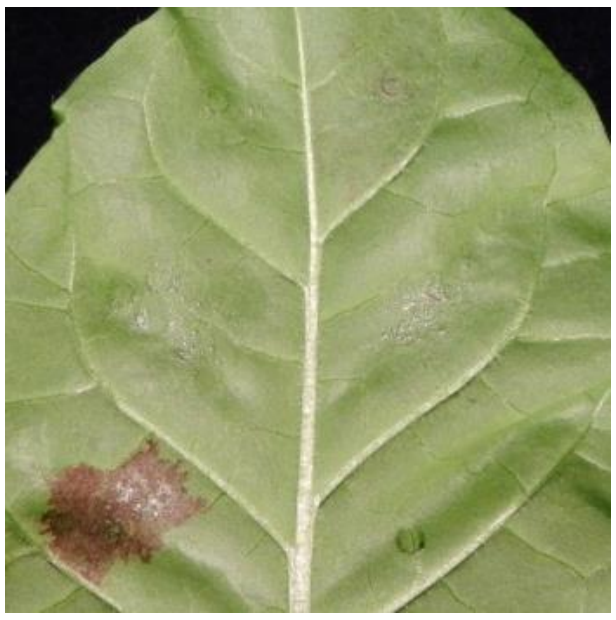

MYB10.1-1k	MYB10.1-1k+PaHLH3
GFP	MYB10.1-1k+GFP
PAP1	MYB10.1-1k+PaHLH33

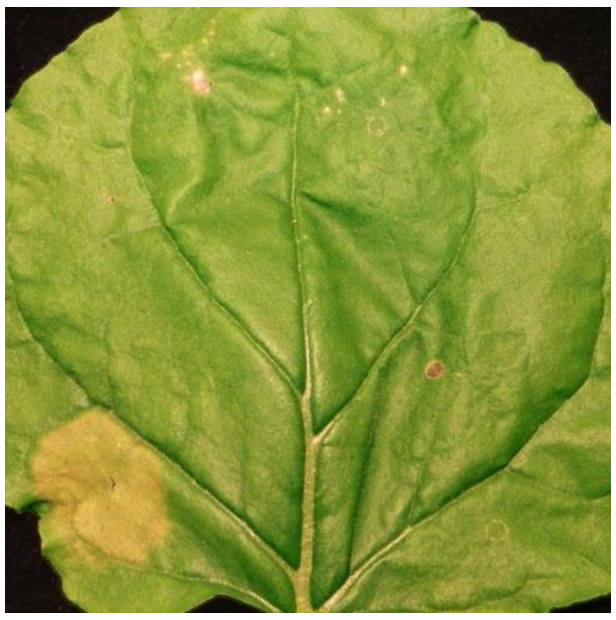

MYB10.1-1	MYB10.1-1+PaHLH3
ANT1*	ANT1*+PaHLH3
PAP1	PAP1+PaHLH3

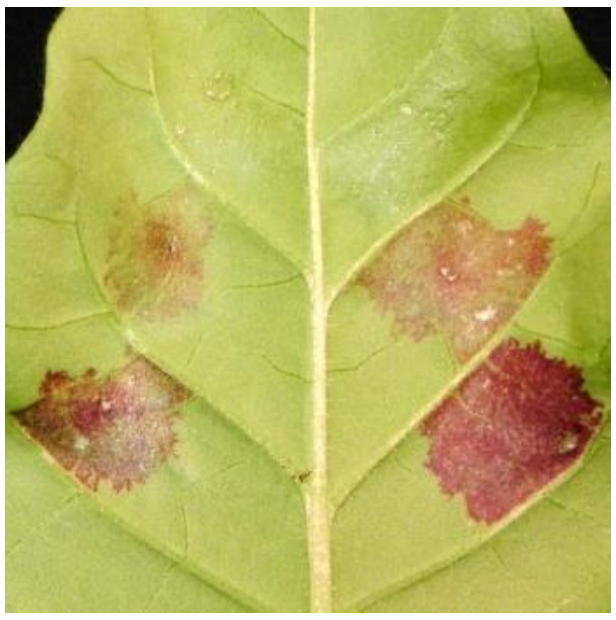

MYB10.1-1k+PaHLH33+PaWD40	MYB10.1-1k+PaHLH33
MYB10.1-1k+PaWD40	MYB10.1-1k
MYB10.1-1k+PaHLH3+PaWD40	MYB10.1-1k+PaHLH3

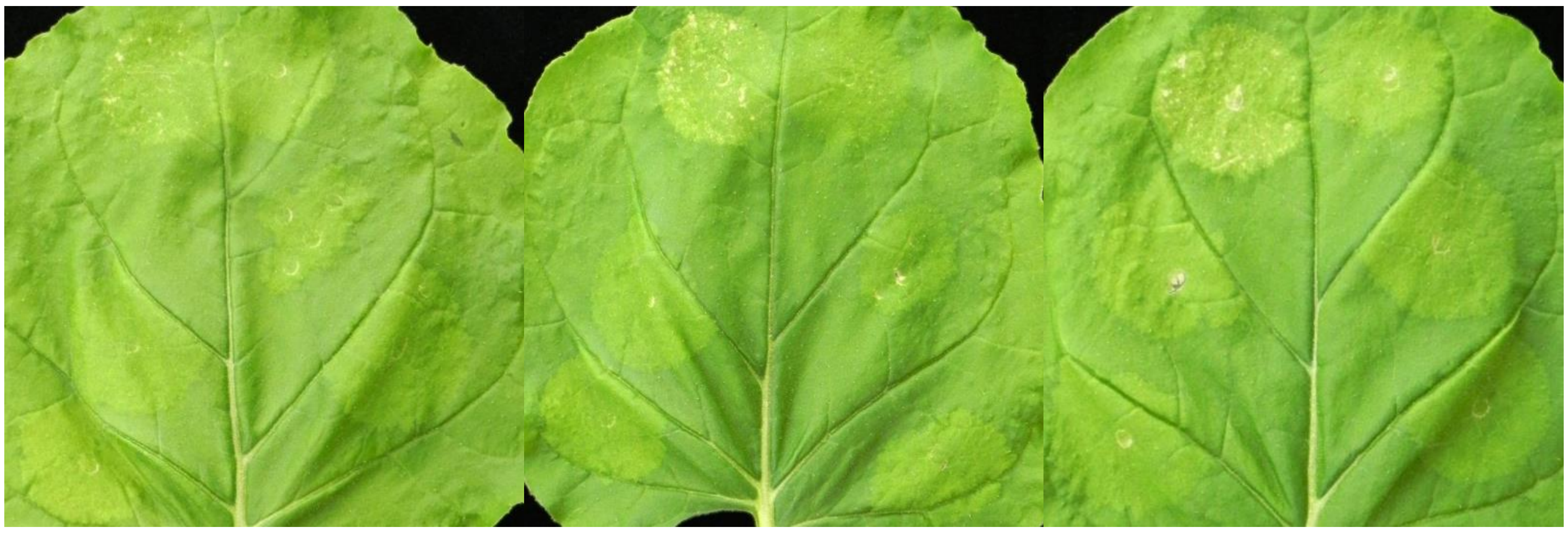

Nicotiana tabacum

Nicotiana benthamiana

PaMYB10.1-2

MYB10.1-2+PaHLH33	MYB10.1-2+PaHLH33+PaWD40
MYB10.1-2	MYB10.1-2+PaWD40
MYB10.1-2+PaHLH3	MYB10.1-2+PaHLH3+PaWD40
GFP+PaHLH3	PAP1+PaHLH3

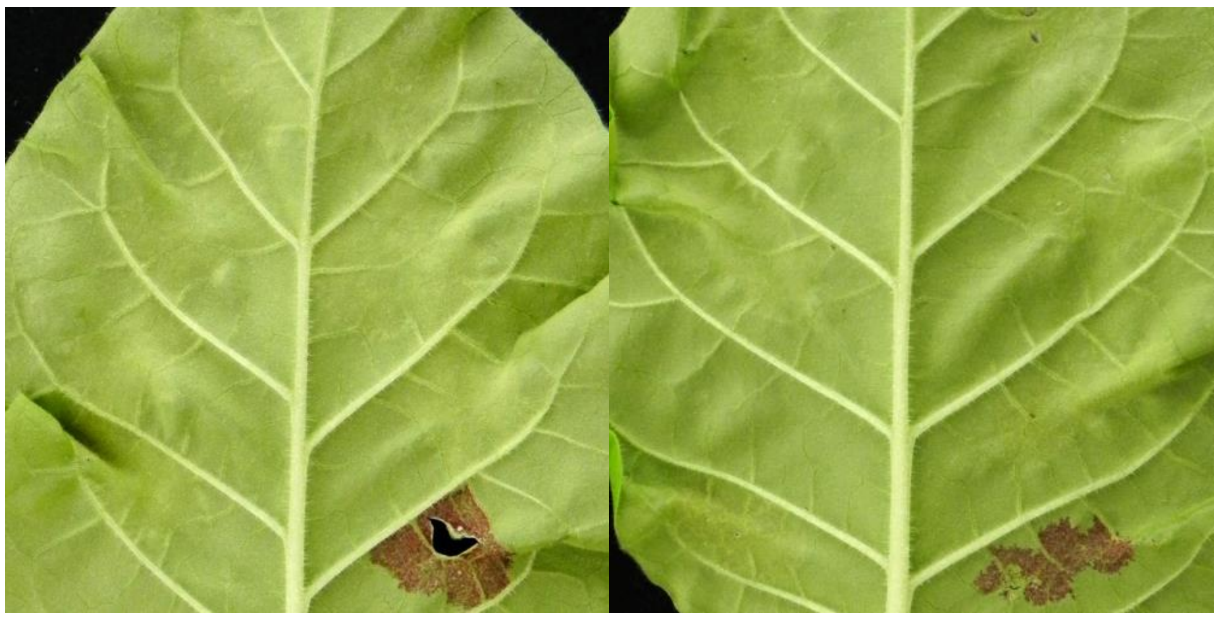

MYB10.1-2+PaHLH33+PaWD40	MYB10.1-2+PaHLH33
MYB10.1-2+PaWD40	MYB10.1-2
MYB10.1-2+PaHLH3+PaWD40	MYB10.1-2+PaHLH3

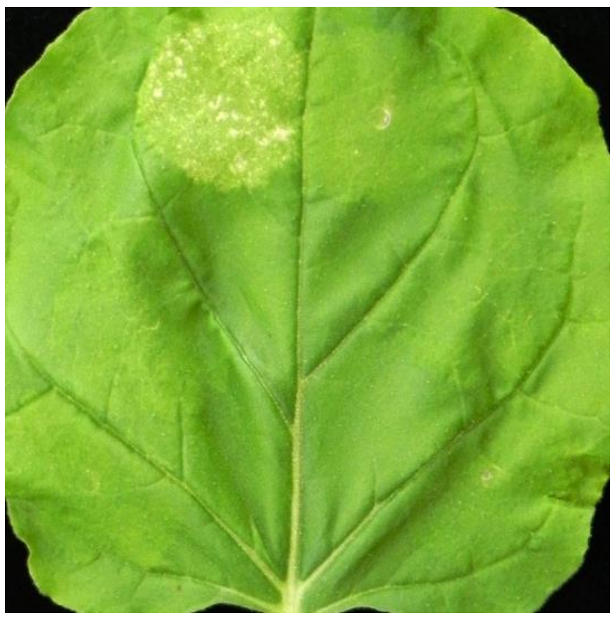

MYB10.1-2	MYB10.1-2+PaHLH3
GFP	MYB10.1-2+GFP
PAP1	MYB10.1-2+PaHLH33

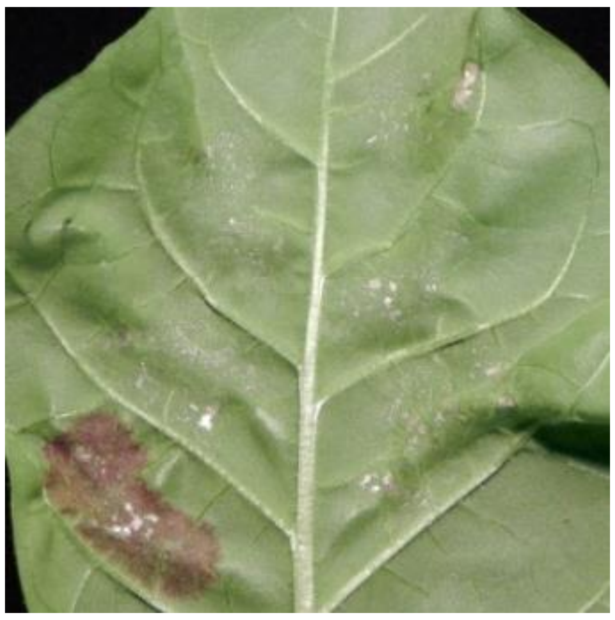

MYB10.1-2	MYB10.1-2+PaHLH3
GFP	MYB10.1-2+GFP
PAP1	MYB10.1-2+PaHLH33

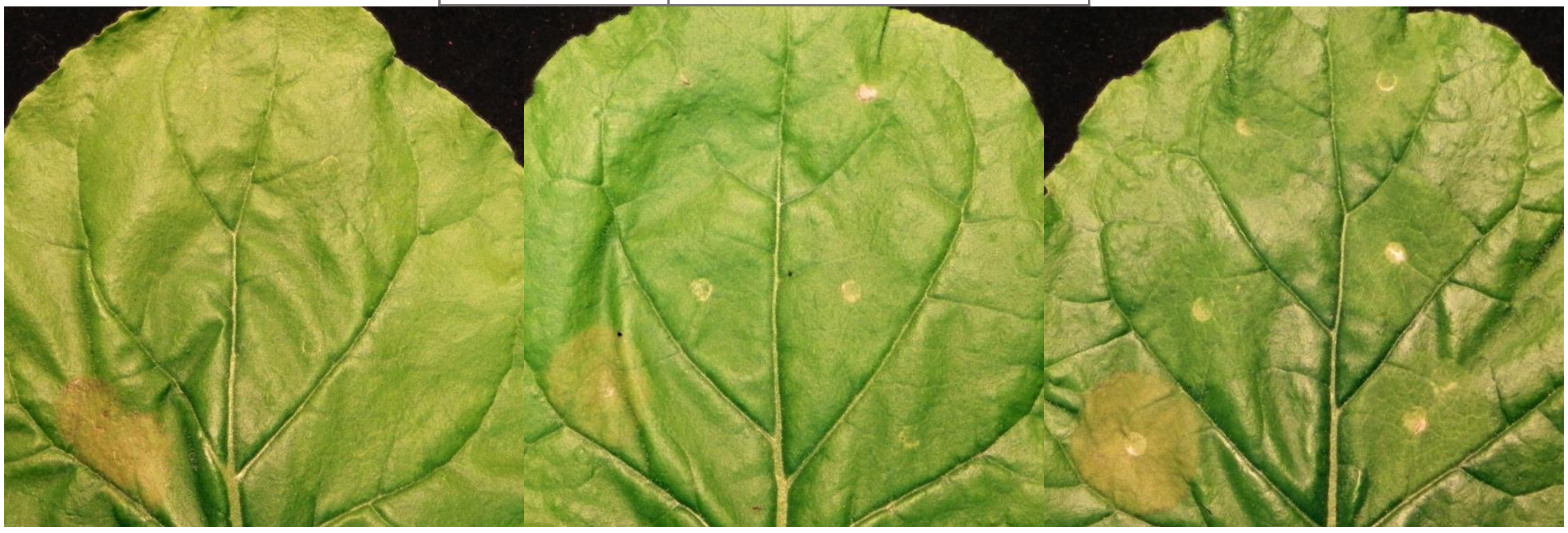

Nicotiana tabacum

Nicotiana benthamiana

PaMYB10.1-3

MYB10.1-3+PaHLH33	MYB10.1-3+PaHLH33+PaWD40
MYB10.1-3	MYB10.1-3+PaWD40
MYB10.1-3+PaHLH3	MYB10.1-3+PaHLH3+PaWD40
ANT1*	PAP1

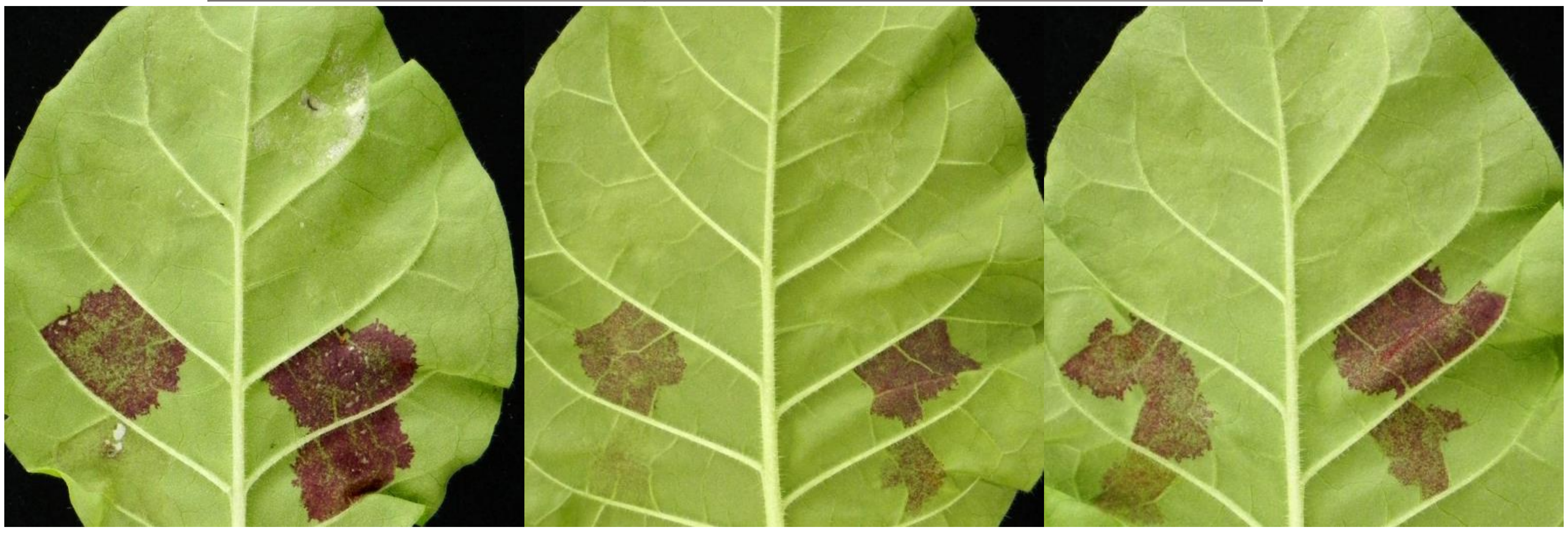

MYB10.1-3+PaHLH33+PaWD40	MYB10.1-3+PaHLH33
MYB10.1-3+PaWD40	MYB10.1-3
MYB10.1-3+PaHLH3+PaWD40	MYB10.1-3+PaHLH3

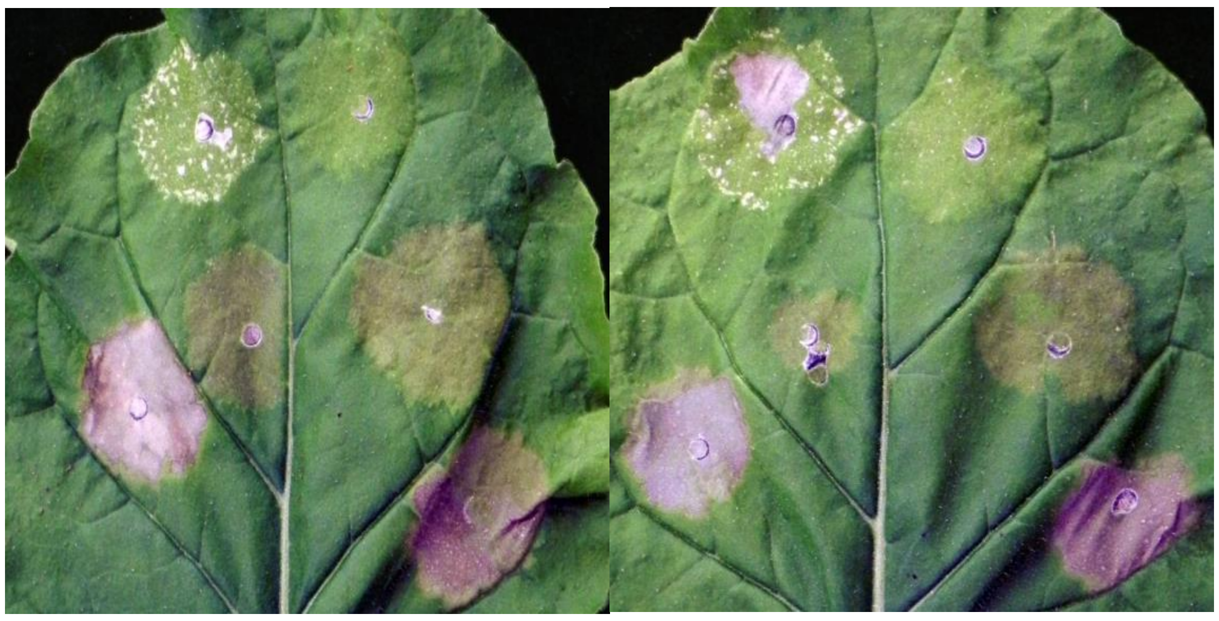

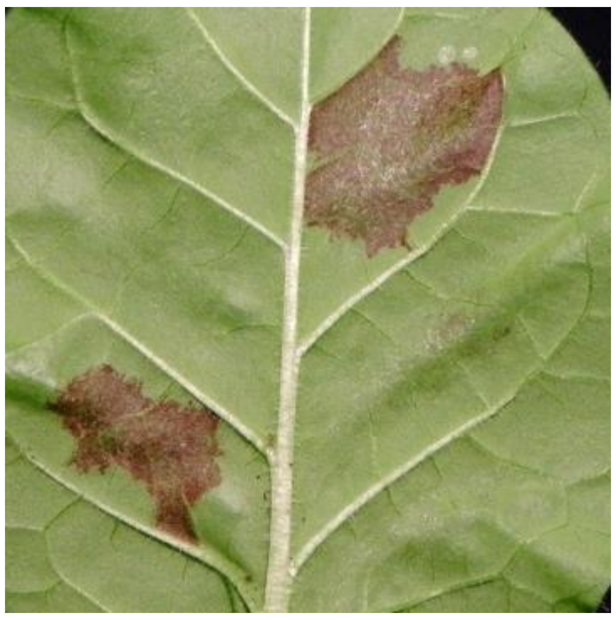

MYB10.1-3	MYB10.1-3+PaHLH3
GFP	MYB10.1-3+GFP
PAP1	MYB10.1-3+PaHLH33

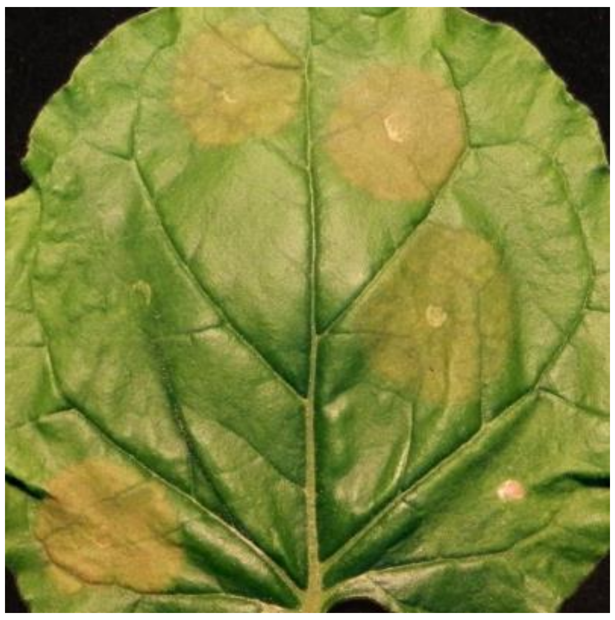

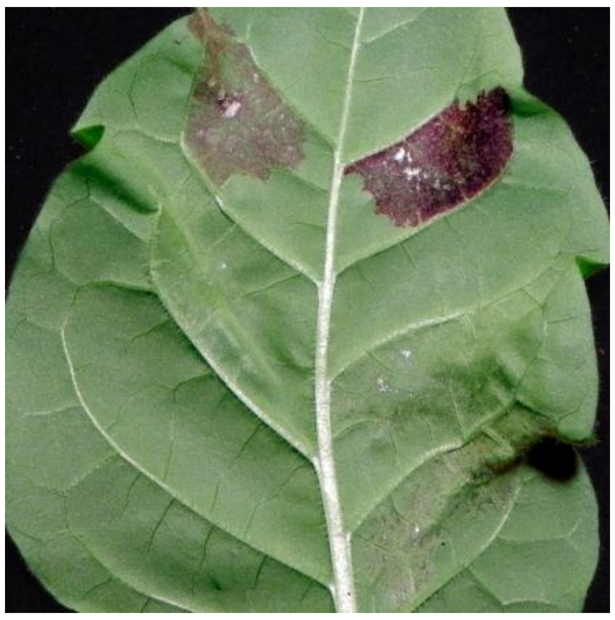

ANT*	ANT*+PAP1
GFP	PaHLH3+PaHLH33
	MYB10.1-3+PaHLH3+PaHLH33

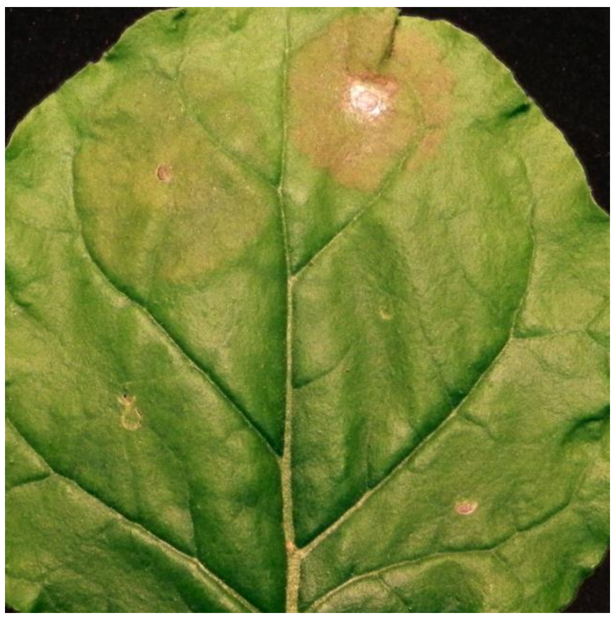

Nicotiana tabacum *Nicotiana benthamiana*

PaMYB1-1 - PaMYB10.1-3 - PaMYB10.1-3k

MYB10.1-3	MYB10.1-3+PaHLH3
MYB10.1-3k	MYB10.1-3k+PaHLH3
PAP1	PAP1+PaHLH3

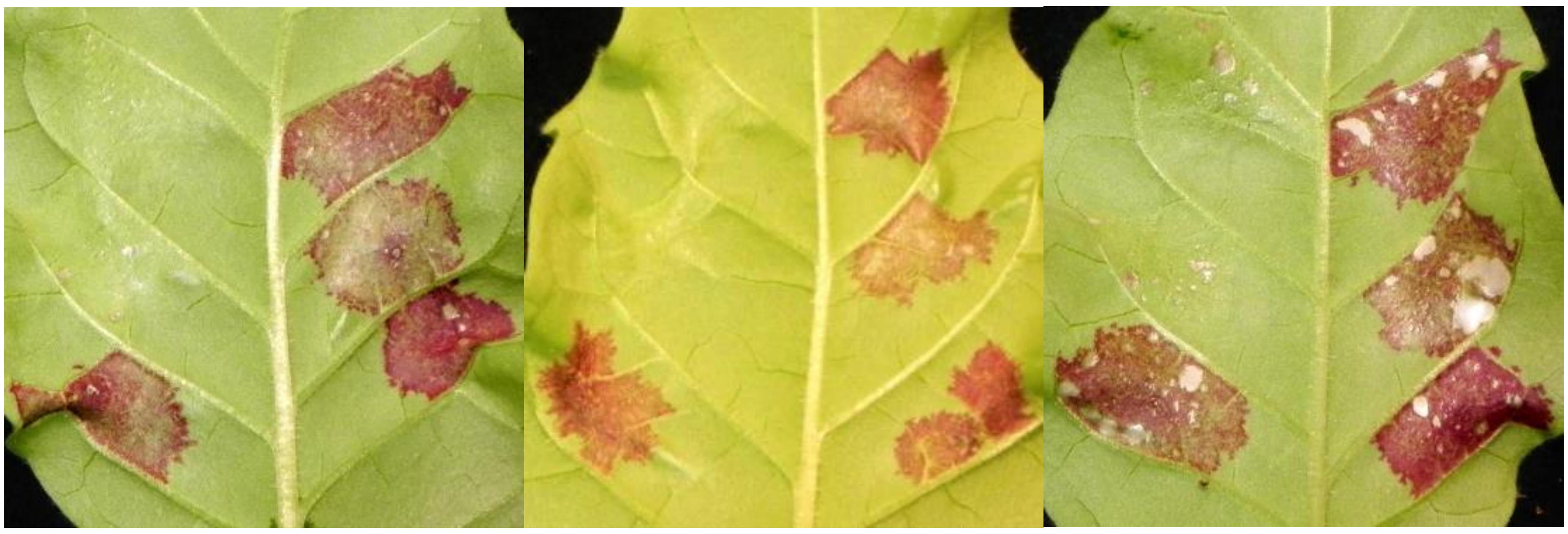

MYB10.1-1	MYB10.1-1+PaHLH3
MYB10.1-3k	MYB10.1-3k+PaHLH3
ANT1*	ANT1*+PaHLH3

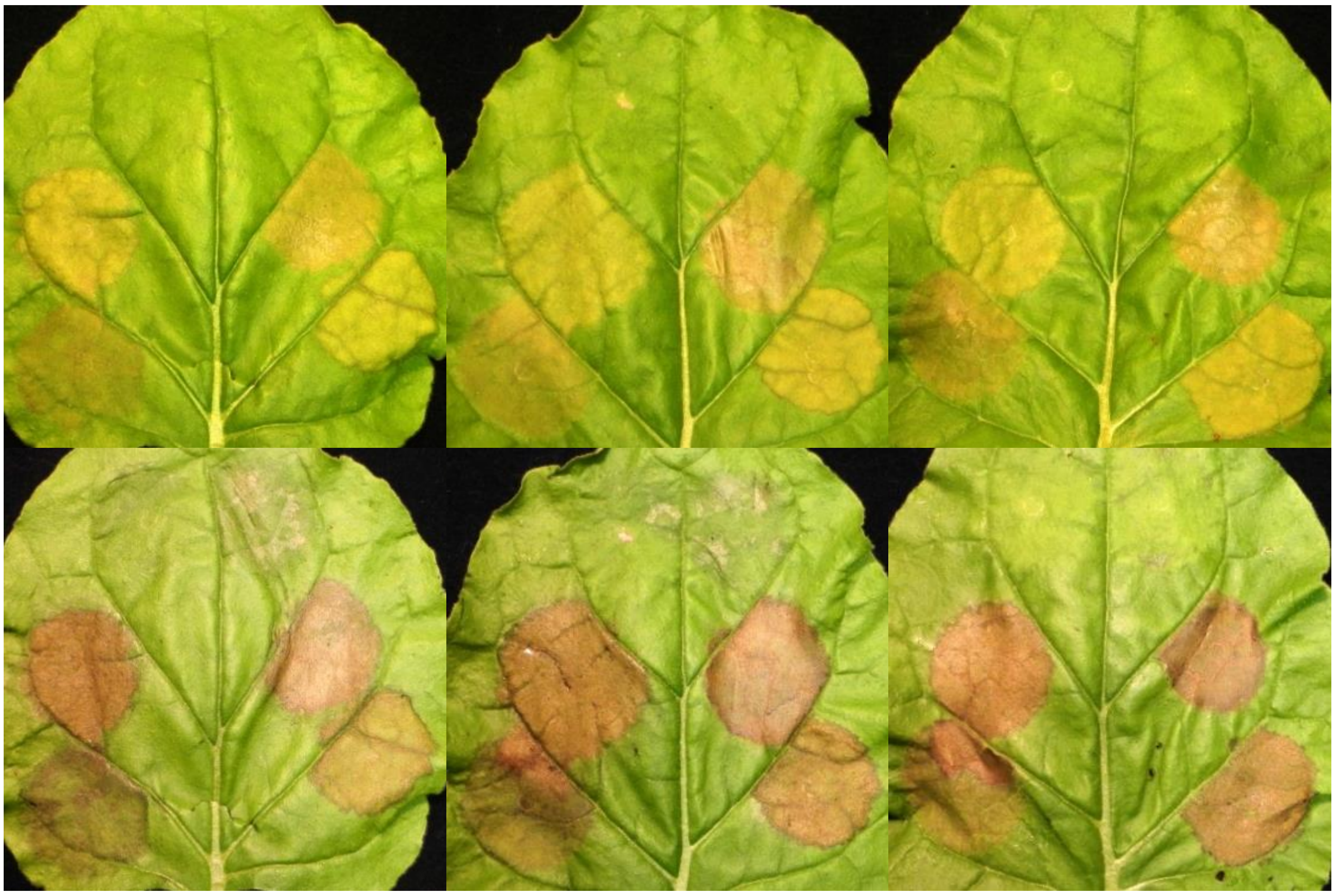

After storage at 7°C

Nicotiana tabacum

Nicotiana benthamiana

PaMYB10.1-3 - PaMYB10.1-3k – PaMYB1-3a

PaHLH3	PaHLH33
GFP	MYB10.1-3+PaWD40
MYB10.1-3+PaHLH3+PaWD40	MYB10.1-3+PaHLH33+PaWD40
MYB10.1-3+PaHLH3	MYB10.1-3+PaHLH33
PAP1	MYB10.1-3

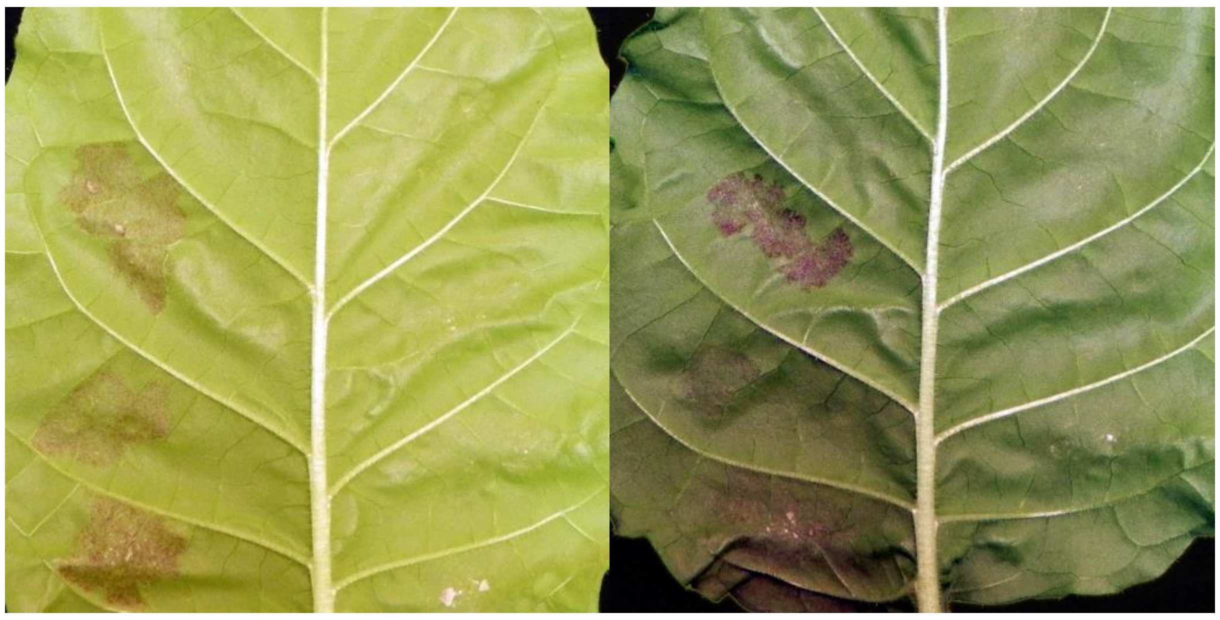

MYB10.1-3a	MYB10.1-3a+PaHLH3

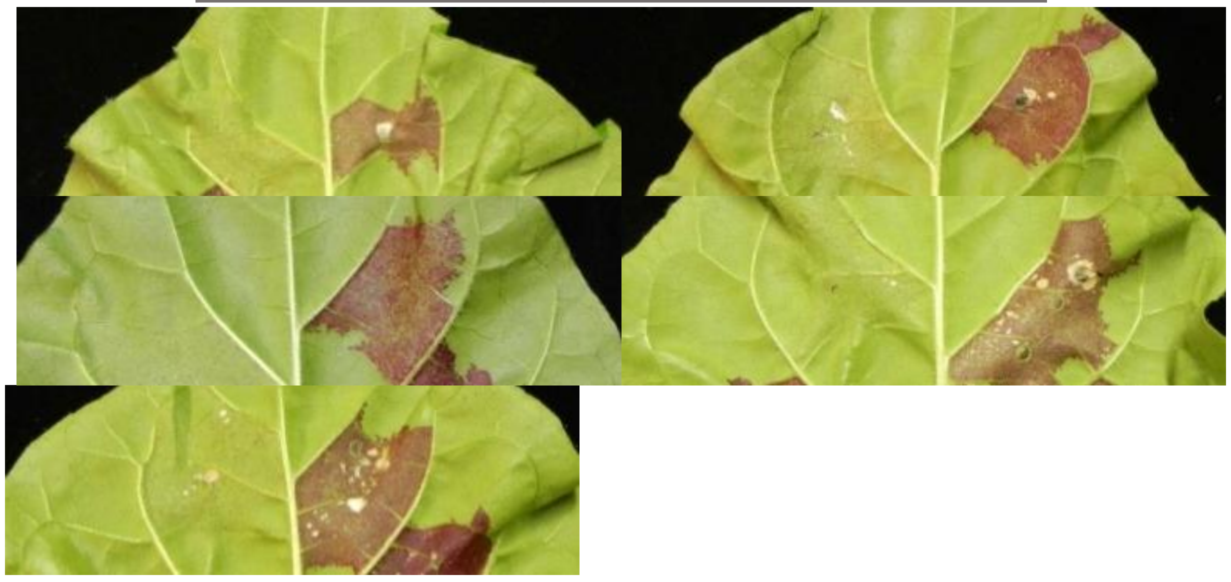

Nicotiana tabacum

GFP	MYB10.1-3k+GFP
	PAP1

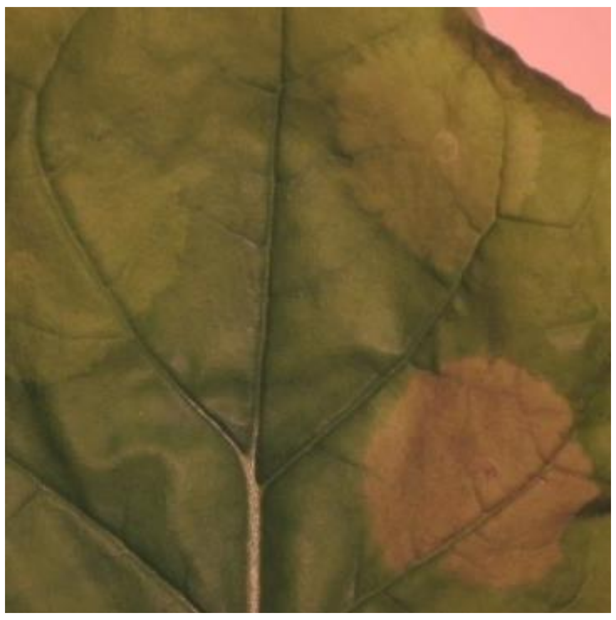

	MYB10.1-3k
MYB10.1-3	PAP1

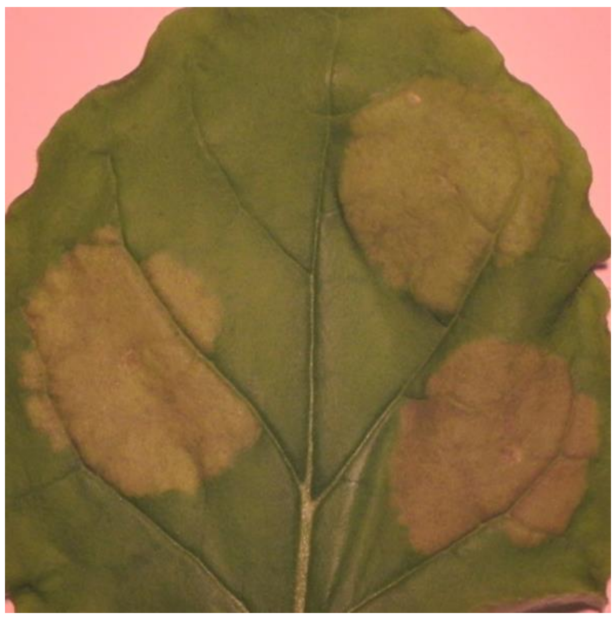

Nicotiana benthamiana
