# Supplementary material for: Expression and Anthocyanin Biosynthesis-Modulating Potential of Sweet Cherry (Prunus avium L.) MYB10 and bHLH Genes
Source: PLoS One. 2015 May 15;10(5):e0126991. doi: 10.1371/journal.pone.0126991 (PMC4433224; doi:10.1371/journal.pone.0126991)
Supplement: S1 Table — (PDF) [file pone.0126991.s004.pdf]

Primers for cloning of genes

Name	Sequence
**For cloning of full-length Myb10 genes**	
PrPro_99-72d	ATCCCTTTATTTATAATGCTAGGTGGCTTC
PrCDS_2223-2196r	CTATTCTTCTTTTGAATGATTCCAAAGG
**Primer for 3'-RACE-ready cDNA synthesis**	
3-RACE-CDS-A	AAGCAGTGGTATCAACGCAGAGTACTTTTTTTTTTTTTTTTTTTTTTTTTTTTTTVN
**Primer for 5'RACE-ready and qPCR cDNA synthesis**	
HOUSE_A	CACGGTCCATCGCAGCAGTCACTTTTTTTTTTTTTTTTTTTTTTTTTTTTTTVN
**Template Switch Oligo**	
SMART-C3	AAGCAGTGGTATCAACGCAGAGTACATGGGG-C3spacer
**Nonspecific (for target gene) primers for 3' and 5'-RACE**	
UPM = UPM-Long-A + UPM-Short-A	
UPM-Long-A	CTAATACGACTCACTATAGGGCAAGCAGTGGTATCAACGCAGAGT
UPM-Short-A	CTAATACGACTCACTATAGGGC
NUP-A	AAGCAGTGGTATCAACGCAGAGT
