# Supplementary material for: Expression and Anthocyanin Biosynthesis-Modulating Potential of Sweet Cherry (Prunus avium L.) MYB10 and bHLH Genes
Source: PLoS One. 2015 May 15;10(5):e0126991. doi: 10.1371/journal.pone.0126991 (PMC4433224; doi:10.1371/journal.pone.0126991)
Supplement: S2 Table — (PDF) [file pone.0126991.s005.pdf]

Primers for qPCR

Direct		Reverse		Target mRNA
Name	Sequence	Name	Sequence	
ACTd	CAACTATGTTCCCCGGTATTGC	ACTr	CCCTTGGAAATCCACATCTGC	Actin V1
PALd	CTACATCGACGACCCCTGC	PALr	CCCTTGCACTATCCACCTCT	
CHSd	GCCCAGCAATTCTAGACCAAG	CHSr	CCCCAATCCAGTCCATCTCC	CHS
CHId	CACTGATGCAGAAGCCAAGG	CHIr	TTGGGAGATTGTGTGAAAAGGA	CHI
F3Hd	GTTCAACCAGTGGAAGGAGC	F3Hr	TGAGTTCACAACTGCTTGGTG	F3H
F3'Hd	TAATGGCTGCAACCCTGGTCC	F3'Hd	GCTTTGTATGCATGAGGGGCTAG	DFR
DFRd	ATGACTGGTTGGATGTACTTCG	DFRr	GATGAGGCTTGGTGGCATG	DFR
ANSd	CCACAACATGGTTCCTGGC	ANSr	CCTTCTCCTTGTTCACCATTCC	ANS
UFGTd	GCCTTCGTAACGCACTGC	UFGTr	ATCTCCAACACGTCCTCCAC	UFGT
WD40d	CTTCGATTTGCGGGACAAGG	WD40r	CCATGTACCTCAGGTCCTGC	WD40
bHLH33d	GCTCACTCAGTCCAATCATCC	bHLH33d	CGCTGCTCCTCGAAACTTTG	bHLH33
bHLH3d	AGAGTGACGGGTTGTTGGAG	bHLH3r	CCCTCAGCTCAGCTACGAAG	bHLH3
V0d	GCCAAATATCAAGAGAGGAGACTTTATG	V2r	ATCATTCGCAGTCCTTCCTGGAAGTCTTTG	MYB10.2 (V0-V0)
V1d	TCAAGATAGGAGGGTTTGCAGAG	V1r	ATCATTCGCTGTCCTTCCTGGAAGCCTTCC	MYB10.1 (V1-V1)
V2d	ATCGTAATAAGACCTCAACCCCA	V2r	CTCGACCGTTTGAATATGGTCC	MYB10.1-1 (V2-V2)
V3d	CCATCATAATAAGACCTCAACCACG	V3r	CTCAACTGTTTGAATATGGGGTTC	MYB10.1-3 (V3-V3)
