# Supplementary material for: Expression and Anthocyanin Biosynthesis-Modulating Potential of Sweet Cherry (Prunus avium L.) MYB10 and bHLH Genes
Source: PLoS One. 2015 May 15;10(5):e0126991. doi: 10.1371/journal.pone.0126991 (PMC4433224; doi:10.1371/journal.pone.0126991)
Supplement: S3 Table — (PDF) [file pone.0126991.s006.pdf]

Primers for construction of plasmids for transient gene expression

Name	Sequence	Gene
**P84f NruI**<br>**P84r NotI**	ATCGCGAATGGAGAACTCAGCACAAG<br>TGCGGCCGCTCAAACCTTCAAAAGC	PaWD40
**P253f PvuII**<br>**P253r NotI**	ACAGCTGATGGCTGCACCGCCAAGCAG<br>AGCGGCCGCCTAGGAATCAGATTGGGGAA	PabHLH3
**P261f NruI**<br>**P261r NotI**	ATCGCGAATGGCCAATGGTACTCAAAACCA<br>AGCGGCCGCTCAACACTTACCGGCAATTT	PabHLH33
**P398f NruI**<br>**P398r NotI**	ATCGCGAATGGAGGGCTATAACTTGGG<br>AGCGGCCGCCTATTCTTCTTTTGAATGAT	PaMYB10.1-2,<br>PaMYB10.1-3
**P398f NruI**<br>**P229r NotI**	ATCGCGAATGGAGGGCTATAACTTGGG<br>AGCGGCCGCCTATTCTTTTTTTAATGATT	PaMYB10.1-1
**ANT1f**<br>**ANT1r**	AGGTCTCACATGAACAGTACATCTATGTCTTCAT<br>AGGTCTCTAAGCTTAATCAAGTAGATTCCATAAGTCAA	SlANT1
**PAP1f SmiI**<br>**PAP1r NotI**	AATTTAAATGGAGGGTTCGTCCAAAGGG<br>AGCGGCCGCTAATCAAATTTCACAGTCTCTCCATC	AtPAP1
